# Benchmark of Density Functionals for the Calculation of the Redox Potential of Fe^3+^/Fe^2+^ Within Protein Coordination Shells

**DOI:** 10.3389/fchem.2019.00391

**Published:** 2019-06-05

**Authors:** Risnita Vicky Listyarini, Diana Sofia Gesto, Pedro Paiva, Maria João Ramos, Pedro Alexandrino Fernandes

**Affiliations:** UCIBIO-REQUIMTE, Departamento de Química e Bioquímica, Faculdade de Ciências, Universidade do Porto, Porto, Portugal

**Keywords:** redox potencial, DFT, benchmaking, iron, quantum-chemical calculations

## Abstract

Iron is a very important transition metal often found in proteins. In enzymes specifically, it is often found at the core of reaction mechanisms, participating in the reaction cycle, more often than not in oxidation/reduction reactions, where it cycles between its most common Fe(III)/Fe(II) oxidation states. QM and QM/MM computational methods that study these catalytic reaction mechanisms mostly use density functional theory (DFT) to describe the chemical transformations. Unfortunately, density functional is known to be plagued by system-specific and property-specific inaccuracies that cast a shadow of uncertainty over the results. Here we have modeled 12 iron coordination complexes, using ligands that represent amino acid sidechains, and calculated the accuracy with which the most common density functionals reproduce the redox properties of the iron complexes (specifically the electronic component of the redox potential at 0 K, $\Delta {\text{E}}_{\text{elec}}^{\text{F}{\text{e}}^{3+}/\text{F}{\text{e}}^{2+}}$), using the same property calculated with CCSD(T)/CBS as reference for the evaluation. A number of hybrid and hybrid-meta density functionals, generally with a large % of HF exchange (such as BB1K, mPWB1K, and mPW1B95) provided systematically accurate values for $\Delta {\text{E}}_{\text{elec}}^{\text{F}{\text{e}}^{3+}/\text{F}{\text{e}}^{2+}}$, with MUEs of ~2 kcal/mol. The very popular B3LYP density functional was found to be quite precise as well, with a MUE of 2.51 kcal/mol. Overall, the study provides guidelines to estimate the inaccuracies coming from the density functionals in the study of enzyme reaction mechanisms that involve an iron cofactor, and to choose appropriate density functionals for the study of the same reactions.

## Introduction

Iron plays an important role in enzyme catalysis. It is present in a wide range of proteins as a cofactor, and intervenes in many biochemical processes, particularly in oxygen transport, and electron transfer. Iron can be often found incorporated in heme prosthetic groups, or it can also occur individually, as an ion cofactor in some metalloproteins, or, more unfrequently, together with sulfide, forming iron-sulfur clusters (Harding et al., 2010; Abbaspour et al., 2014).

One of the most important characteristics of iron is the fact that it can be found in two stable states of oxidation, Fe^2+^ and Fe^3+^. It is this characteristic that allows iron to participate in a wide range of redox reactions (Broderick, 2001). In proteins, it is usually found in the active site, coordinated with the side chains of amino acid residues and/or water molecules. Interestingly, the redox potential of the iron ion changes depending on the ligands it is complexed with. In enzymes, through evolution, the ligands to which the iron is coordinated were selected so that its redox potential became the most appropriate for the reaction catalyzed by it (Tamames and Ramos, 2010; Liu et al., 2014).

Iron is of extreme importance to almost all organisms. In adult humans, iron makes up about 0.005% of the total body weight (circa 4 g), most of which is complexed with the heme group in hemoglobin. Iron is also present in cytochromes, heme-containing proteins that are involved in many electron transfer reactions that occur in our body, from oxidative phosphorylation to the synthesis of hormones or the degradation of drugs (Abbaspour et al., 2014).

The reduction potential (or redox potential) of an atom/molecule is, by definition, a measurement of its ability to gain an electron, and therefore be reduced (Equation 1).

(1) $$\begin{array}{r} M^{n}\;+\;e^{-}\;\to \;M^{n-1} \end{array}$$

The reduction potential is not a fixed value for a given chemical entity, and can change depending on different factors, such as temperature, pH, structure, conformation, and electrostatic environment (Su et al., 2011; Ho et al., 2016). For this reason, the redox potentials of metal cofactors need to be calculated for each enzyme individually. In proteins, iron acts as both an electron donor and acceptor, depending on its oxidation state. When it is in the form of Fe(II), iron will mostly give up one of its electrons, thus being oxidized to Fe(III). On the other hand, when in the form of Fe(III), it can act as a oxidizing agent, gaining one electron, and changing its oxidation state to Fe(II). In some rarer cases, it is possible to find Fe^4+^ as a cofactor of some enzymes as well, but these cases will not be addressed in this paper.

Since iron is such an important metal in biological processes, with its redox potential changing greatly depending on the ligands coordinated to it, the study of these processes, and the accuracy of the final results, is also determined by how well we understand the redox chemistry of the iron ion. The measurement of reduction potentials in biological systems using experimental methodologies is a very complex and difficult task. The alternative is the use of high-level computational methods, which employ elaborate algorithms, equations, and approximations to simulate the system in question and achieve the most accurate result possible (Riley and Merz, 2007). However, it should be noted that in order to obtain these results one must employ methods that are extremely time consuming and CPU demanding, which is impractical for biological systems such as proteins, where the number of atoms is exceedingly high. It is possible to use less accurate methods when calculating redox potentials in such systems, but the problem in these cases is that, due to their limited accuracy, we might not know which one is the best for each chemical scenario. To solve this problem, we can benchmark the different methods, and compare these results to a reference value, ranking each method as more or less accurate.

The main focus of this work is, therefore, to perform a benchmark study of different computational methods and evaluate their ability to simulate the iron ion in different arrangements, often found in proteins. We selected only density functional theory (DFT) (Thomas, 1927; Hohenberg and Kohn, 1964) methods because of their very good accuracy and applicability on large systems (they have a more favorable scaling with the system size than post-Hartree Fock methods). DFT methods are a good choice when it comes to simulating proteins (Uudsemaa and Tamm, 2003; Riley et al., 2007; Li et al., 2009). The problem comes when selecting which density functional to use for a specific case, since their performance can differ depending on the reaction, model, chemical system, etc. For this reason, benchmarking studies are gaining increasingly more attention, as they give us the ability to make a more informed decision on which DFT methods might be more accurate for the system we want to study.

For this benchmark study, in order to mimic the many complexes that are possible for an iron ion to have in the protein, we constructed 12 models, that span over different coordination numbers and ligands. The models try to mirror the various types of amino acid side chains (and water molecules) that can be most frequently found coordinating iron in proteins. Only one side chain was used in each model, to evaluate the individual effect of each side chain in the redox potential, albeit in proteins the iron coordination shell is made of many (up to six) side chains simultaneously. The coordination shells were completed with water molecules. Water was treated as a coordinating ligand as well.

For each of these models, we calculated the electronic component of the redox potential at 0 K ($\Delta {\text{E}}_{\text{elec}}^{\text{F}{\text{e}}^{3+}/\text{F}{\text{e}}^{2+}}$) using the different density functionals. We decided to measure only this component, instead of calculating the redox potential in its entirety for several reasons: this electronic component is the one that contributes the most for the overall potential; it is the only component that can be calculated with CCSD(T), which is the method to be used as a reference; it is the component in which the different density functionals show a larger variation in the final results. Furthermore, calculating only $\Delta {\text{E}}_{\text{elec}}^{\text{F}{\text{e}}^{3+}/\text{F}{\text{e}}^{2+}}$ is much easier and faster computationally, and the introduction of the other components in the calculation would render this study much more time consuming with not much improvement in the final results (Su et al., 2011; Ho et al., 2016).

Reference values for $\Delta {\text{E}}_{\text{elec}}^{\text{F}{\text{e}}^{3+}/\text{F}{\text{e}}^{2+}}$, calculated in each system to benchmark the accuracy of the density functionals, were calculated using two post-Hartree-Fock methods: the second order Moller-Plesset perturbation theory (MP2) method, and the single and double coupled cluster theory with perturbative triple correction [CCSD(T)]. The extrapolation energy at the complete basis set (CBS) limit was calculated using two different, widely used extrapolation schemes.

## Computational Methods

### Model Systems and Structure Optimization

The iron complexes found in proteins are quite diversified. Iron can make complexes with a different number of ligands, and, depending on these, the geometry of the complex will also change. Furthermore, the side chains of the amino acid residues that interact and complex with iron have different functional groups.

**Figure 1:**
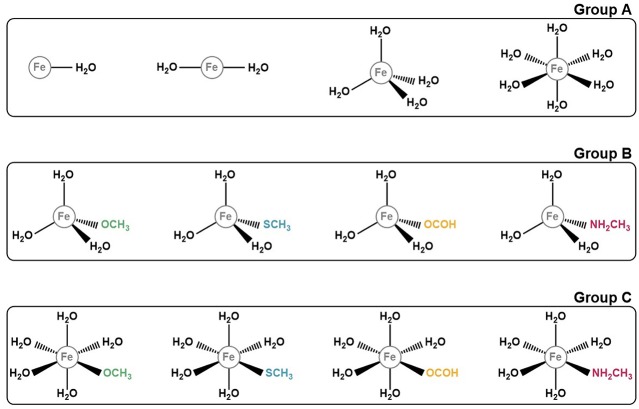
Representation of the 12 iron complexes studied. They were divided in 3 groups according to their ligands for simplicity and for a better discussion. Group A comprises those structures which only have water molecules coordinated to the iron ion. Group B and C include the models with in which one of the ligands mimics an amino acid side chain. The difference between these two groups is their geometry: tetrahedral for group B and octahedral for group C.

In order to take all of these conditions into account in the benchmark, we decided to build 12 model systems, each mimicking a different coordination sphere. These conditions address most of the various coordination states, geometries and ligands that iron complexes can adopt. Our models comprise structures with one, two, four, or six coordinated ligands, which assume geometries of the type linear (for coordination numbers one and two), tetrahedral (for coordination number four), and octahedral (for coordination number six). As for the ligands, we first started with a set of structures in which the iron ion was coordinated exclusively to water molecules, having modeled one structure for each coordination number, with a total of four models. As for the remaining 8, we substituted one of the coordinated waters by one of the following ligands: methoxide (CH_3_O^−^), formate (HCOO^−^), methanethiolate (CH_3_S^−^), and methylamine (NH_2_CH_3_). This choice of ligands reflects our objective of benchmarking the DFT methods in the context of proteins. Each of these entities represents the side chains of one amino acid that frequently participates in the formation of iron complexes in proteins: CH_3_O^−^ mimics the side chain of serine, CHOO^−^ that of glutamate and aspartate, CH_3_S^−^ the side chain of cysteine and NH_2_CH_3_ that of lysine. The structures for all the models studied are represented on Figure 1.

After building each of the models, we optimized their geometries using the MP2/6-311+G(d,p) level of theory. One might argue that, for the sake of consistency, it would have been better to calculate $\Delta {\text{E}}_{\text{elec}}^{\text{F}{\text{e}}^{3+}/\text{F}{\text{e}}^{2+}}$ for each model using the minimum-energy geometries obtained with each functional we purpose to study. In reality, such study would demand a lot more computational time, for a minimal difference in the final results, as shown in previous studies (Ribeiro et al., 2010; Brás et al., 2011).

Furthermore, MP2 geometries are known to give good results without introducing biases toward any functional. All calculations were performed using the Gaussian 09 package (Frisch et al., 2009).

We optimized the geometries of each complex with both Fe(II) and Fe(III) oxidation states. It is known that, for each oxidation state, the iron ion can have two different spin states. For Fe(II), the high spin multiplicity is five and the low spin multiplicity is one. In the case of Fe(III), the high spin multiplicity is six, and the low one is two. We calculated which of these spin states gave the lowest energy for each oxidation state, and used that spin multiplicity for the remainder of our calculations. In both cases, the high spin multiplicity [five for Fe(II) and six for Fe(III)] was the one that resulted in the lowest energies (Riley and Merz, 2007) (data not shown). As a result, these were the spin states used throughout the following calculations. The <S^2^> values in each MP2 calculation were checked and compared to the expected values of 6.0 for Fe(II) complexes and 8.75 for Fe(III) complexes. The Gaussian 09 package includes an annihilation step to decrease the amount of spin contamination. For the Fe(II) complexes, we observed an average <S>^2^ of 6.0079 and 6.0000 before and after annihilation, respectively. Regarding the Fe(III) complexes, the average <S^2^> before and after the annihilation step were 8.7625 and 8.7501, respectively. Thus, the spin contamination is virtually null.

All calculations were made in gas-phase. As the purpose is to benchmark only and specifically the DFs, we avoided to introduce solvation, so that small differences in solvation would not introduce an unwanted contribution for differences in accuracy of the DFs that are being compared.

### Calculation of the Reference $\Delta {\text{E}}_{\text{elec}}^{\text{F}{\text{e}}^{3+}/\text{F}{\text{e}}^{2+}}$ Values

In order to correctly benchmark and compare the diverse density functionals, we needed to obtain accurate values for $\Delta {\text{E}}_{\text{elec}}^{\text{F}{\text{e}}^{3+}/\text{F}{\text{e}}^{2+}}$ in each of our models.

For this study, we decided to calculate $\Delta {\text{E}}_{\text{elec}}^{\text{F}{\text{e}}^{3+}/\text{F}{\text{e}}^{2+}}$ using the very accurate CCSD(T) method (Bartlett and Purvis, 1978; Purvis and Bartlett, 1982; Pople et al., 1987), extrapolated to the CBS limit, and use these values as a reference. To this end, we carried out single point calculations using the MP2/aug-cc-pVXZ (X = 2, 3, 4) and CCSD(T)/aug-cc-pVDZ levels, in vacuum, for each optimized structure, and then we employed two different and well-known extrapolation methods to obtain the final values: one developed by Truhlar (1998) (scheme I) and another by Helgaker et al. (1997), Halkier et al. (1998) (scheme II). Both schemes begin with the extrapolation of the correlation energy to the CBS limit at the MP2 level. The difference between the correlation energies calculated using MP2 and CCSD(T) at the largest basis set possible (for this study, we used aug-cc-pVDZ) is then determined and added to the MP2/CBS value. The extrapolation of the Hartree-Fock (HF) energy is done separately from that of the correlation energy in both schemes as well.

To determine the MP2/CBS energy using scheme I, we employ Equation 2, using the results obtained from the calculations using MP2/aug-cc-pVDZ and MP2/aug-cc-pVTZ. α and β are constants taken from the literature (α = 4.93 and β = 2.13).

(2) $$\begin{array}{r} E_{\frac{MP2}{CBS}}=\frac{3^{\alpha }}{3^{\alpha }-2^{\alpha }}E_{\frac{HF}{TZ}}-\frac{2^{\alpha }}{3^{\alpha }-2^{\alpha }}E_{\frac{HF}{DZ}}+\frac{3^{\beta }}{3^{\beta }-2^{\beta }}E_{\frac{corr}{TZ}}-\frac{2^{\beta }}{3^{\beta }-2^{\beta }}E_{\frac{corr}{DZ}} \end{array}$$

As for scheme II, $E_{\frac{HF}{CBS}}$ is obtained by exponentially fitting the values obtained for the aug-cc-pVDZ, aug-cc-pVTZ, and aug-cc-pVQZ basis sets. $E_{\frac{corr}{CBS}}$ is calculated with Equation 3.

(3) $$\begin{array}{r} E_{\frac{corr}{CBS}}=\frac{4^{3}E_{\frac{corr}{QZ}}-3^{3}E_{\frac{corr}{TZ}}}{4^{3}-3^{3}} \end{array}$$

The HF energy is taken from Equation 2, whichever the scheme is used to extrapolate the correlation energy.

### Benchmarking of the Density Functionals

We selected a set of 44 density functionals and tested their performance against the values for $\Delta {\text{E}}_{\text{elec}}^{\text{F}{\text{e}}^{3+}/\text{F}{\text{e}}^{2+}}$ obtained at the CCSD(T)/CBS level. This selection includes functionals belonging to different classes: 2 local density approximation (LDA) functionals, 7 generalized gradient approximation (GGA), 5 meta-GGA (m-GGA), 12 hybrid-GGA (h-GGA), 10 hybrid-meta-GGA (hm-GGA), 4 double hybrid-GGA (hh-GGA), 1 non-separable gradient approximation (NGA), 1 hybrid-meta-NGA (hm-NGA), 1 meta-NGA (m-NGA), and 1 generalized gradient exchange (GGE) (Table 1). All of the benchmarking calculations were carried out using 6-311+G(2df,2p) basis set, which was selected due to the fact that, for large biological systems, it is the most CBS that can be generally used. Larger basis sets would render the calculations too difficult, making them extremely computationally demanding and time consuming. It is also worth noting that this basis set is frequently close to the DFT CBS limit, and any differences in relative energy (activation energy, reaction energy, redox potentials, etc.) from larger basis sets are usually in the tenths of a kilocalorie per mole. Another reason for the choice of the 6-311+G(2df,2p) basis set was because it is sufficiently large that it minimizes basis set truncation error within DFT, and so we can more accurately evaluate the performance of the functional without the interference from the basis set.

**Table 1:** List of all the density functionals tested in this wok.

**LDA**	**GGA**	**h-GGA**	**hh-GGA**
SPW91 (Hohenberg and Kohn, 1964; Kohn and Sham, 1965; Slater and Phillips, 1974; Perdew, 1986a; Perdew et al., 1991, 1992, 1996a)	BP86 (Perdew, 1986b; Becke, 1988)	B1LYP (Becke, 1988; Lee et al., 1988; Adamo and Barone, 1997)	B2GPPLYP (Becke, 1988; Lee et al., 1988; Grimme, 2006; Karton et al., 2008)
SVWN (Hohenberg and Kohn, 1964; Kohn and Sham, 1965; Slater and Phillips, 1974; Vosko et al., 1980)	BPBE (Becke, 1988; Perdew et al., 1996b)	B3LYP (Becke, 1988, 1996; Lee et al., 1988)	B2PLYP (Becke, 1988; Lee et al., 1988; Grimme, 2006)
**hm-GGA**	BPW91 (Becke, 1988; Perdew et al., 1991, 1992, 1993, 1996a; Burke et al., 1998)	B3P86 (Perdew, 1986b; Becke, 1988, 1996)	DSD-BLYP (Becke, 1988; Lee et al., 1988; Grimme, 2006; Kozuch et al., 2010)
BB1K (Becke, 1988, 1996; Zhao et al., 2004)	G96LYP (Lee et al., 1988; Gill, 1996; Adamo and Barone, 1998a)	B3PW91 (Becke, 1988, 1996; Perdew et al., 1991, 1992, 1993, 1996a; Burke et al., 1998)	mPW2PLYP (Lee et al., 1988; Perdew et al., 1991, 1992, 1993, 1996a; Adamo and Barone, 1998b; Burke et al., 1998; Schwabe and Grimme, 2006)
BMK (Boese and Martin, 2004)	HCTH407 (Hamprecht et al., 1998)	B97-1 (Hamprecht et al., 1998)	**NGA**
M05 (Zhao et al., 2005)	OLYP (Lee et al., 1988; Handy and Cohen, 2001; Hoe et al., 2001)	B97-2 (Wilson et al., 2001)	N12 (Peverati and Truhlar, 2012a)
M05-2X (Zhao et al., 2006; Zhao and Truhlar, 2008)	OPL(Perdew and Zunger, 1981; Handy and Cohen, 2001; Hoe et al., 2001)	B97-D3 (Grimme et al., 2011)	**m-NGA**
M06 (Zhao and Truhlar, 2007)	**m-GGA**	BhandH (Becke, 1988, 1993; Lee et al., 1988)	MN12-L (Peverati and Truhlar, 2012b)
M06-2X (Zhao and Truhlar, 2007)	M06-L (Zhao et al., 2006)	mPW1K (Perdew et al., 1991, 1992, 1993, 1996a; Adamo and Barone, 1998b; Burke et al., 1998; Lynch et al., 2000)	**hm-NGA**
M11 (Peverati and Truhlar, 2011a)	M11-L (Peverati and Truhlar, 2011b)	mPW1N (Perdew et al., 1991, 1992, 1993, 1996a; Adamo and Barone, 1998b; Burke et al., 1998; Kormos and Cramer, 2002)	MN12-SX (Peverati and Truhlar, 2012b,c)
mPW1B95 (Perdew et al., 1991, 1992, 1993, 1996a; Becke, 1996; Adamo and Barone, 1998b; Zhao and Truhlar, 2004)	mPWB95 (Perdew et al., 1991, 1992, 1993, 1996a; Becke, 1996; Adamo and Barone, 1998b)	PBE1PBE (Perdew et al., 1996b; Adamo and Barone, 1997)	**GGE**
mPWB1K (Perdew et al., 1991, 1992, 1993, 1996a; Becke, 1996; Adamo and Barone, 1998b; Boese et al., 2003; Zhao and Truhlar, 2004)	OTPSS (Handy and Cohen, 2001; Hoe et al., 2001; Tao et al., 2003)	wB97X-D (Chai and Head-Gordon, 2008)	OVWN5 (Vosko et al., 1980; Handy and Cohen, 2001; Hoe et al., 2001)
TPSSh (Tao et al., 2003)	VSXC (Van Voorhis and Scuseria, 1998)		

In order to calculate $\Delta {\text{E}}_{\text{elec}}^{\text{F}{\text{e}}^{3+}/\text{F}{\text{e}}^{2+}}$ with each of the DFT methods, we used the structures optimized at the MP2/6-311+G(d,p) level of theory, for both Fe(II) and Fe(III) and determined their energies through single point calculations. $\Delta {\text{E}}_{\text{elec}}^{\text{F}{\text{e}}^{3+}/\text{F}{\text{e}}^{2+}}$ was then calculated from the difference between each Fe(II)/Fe(III) pair for the corresponding complex. All DFT calculations were carried out using the Gaussian 09 suite (Frisch et al., 2009).

## Results and Discussion

### Calculation of the Electronic Component of the Reduction Potential at the MP2/CBS Level

In order to obtain an accurate measure of $\Delta {\text{E}}_{\text{elec}}^{\text{F}{\text{e}}^{3+}/\text{F}{\text{e}}^{2+}}$ for each iron complex, after the initial geometry optimization of the complexes we used high-level *ab initio* post-HF methods, with CBS extrapolation to eliminate any basis set truncation error. This was done by calculating these values first with the MP2 method, with aug-cc-pVXZ (X = 2, 3, and 4) with subsequent extrapolation to the MP2/CBS level. Extrapolation to this level was done using two different schemes, as described in the methods section of this paper. These values were used in conjunction with the CCSD(T) results (at the corresponding basis set level) in order to extrapolate the energies to the CCSD(T)/CBS level. In this manner, we ensure that we have reliable values for $\Delta {\text{E}}_{\text{elec}}^{\text{F}{\text{e}}^{3+}/\text{F}{\text{e}}^{2+}}$ for each of our 12 models, and that these values can be used as a reference to ascertain the precision of various density functionals in calculating the electronic energy contribution of Fe(II) and Fe(III) in each system. Table 2 summarizes the results obtained for $\Delta {\text{E}}_{\text{elec}}^{\text{F}{\text{e}}^{3+}/\text{F}{\text{e}}^{2+}}$ at the MP2 level and the aug-cc-pVDZ, aug-cc-pVTZ, and aug-cc-pVQZ basis sets.

**Table 2:** Results for $\Delta {\text{E}}_{\text{elec}}^{\text{F}{\text{e}}^{3+}/\text{F}{\text{e}}^{2+}}$ calculated at the MP2 level and the aug-cc-pVDZ, aug-cc-pVTZ, and aug-cc-pVQZ basis sets, for each model.

**Model**	$\Delta {\text{E}}_{\text{elec}}^{{\text{Fe}}^{3+}/{\text{Fe}}^{2+}}$ **(kcal/mol)**		
	**MP2/aug-cc-pVDZ**	**MP2/aug-cc-pVTZ**	**MP2/aug-cc-pVQZ**
Fe(H_2_O)	−596.6545	−599.1266	−601.7583
Fe(H_2_O)_2_	−532.2910	−534.6568	−537.1668
Fe(H_2_O)_4_	−436.5451	−438.9066	−441.2415
Fe(H_2_O)_6_	−381.3428	−383.5432	−385.8931
Fe(H_2_O)_3_(CH_3_O^−^)	−271.8354	−274.9105	−276.9331
Fe(H_2_O)_3_(CH_3_S^−^)	−258.7914	−261.2471	−262.7309
Fe(H_2_O)_3_(NH_2_CH_3_)	−418.7231	−421.6494	−423.9952
Fe(H_2_O)_3_(HCOO^−^)	−300.7855	−303.4670	−305.5606
Fe(H_2_O)_5_(CH_3_O^−^)	−246.0797	−248.7022	−250.6648
Fe(H_2_O)_5_(CH_3_S^−^)	−236.5792	−238.9616	−240.4693
Fe(H_2_O)_5_(NH_2_CH_3_)	−370.4211	−373.1151	−375.4922
Fe(H_2_O)_5_(HCOO^−^)	−269.0788	−271.3712	−273.4479

$\Delta {\text{E}}_{\text{elec}}^{\text{F}{\text{e}}^{3+}/\text{F}{\text{e}}^{2+}}$changed by 2–3 kcal/mol when moving from aug-cc-pVDZ to the aug-cc-pVTZ basis set, and by a further ~2–3 kcal/mol when moving from aug-cc-pVTZ to the aug-cc-pVQZ basis set, emphasizing the need for the extrapolation for the CBS limit. As stated previously, we used two different schemes to determine the MP2/CBS energies. The first extrapolation scheme we used was scheme I, developed by D. Truhlar. Table 3 shows the values obtained at the CBS limit using this scheme, as well as the differences between these energies and the corresponding ones at the aug-cc-pVXZ (X = 2, 3, and 4) basis sets.

**Table 3:** $\Delta {\text{E}}_{\text{elec}}^{\text{F}{\text{e}}^{3+}/\text{F}{\text{e}}^{2+}}$obtained at the MP2/CBS level, extrapolated using scheme I, for each model, and the differences between these new energies and the ones obtained in Table 2.

**Model**	$\Delta {\text{E}}_{\text{elec}}^{{\text{Fe}}^{3+}/{\text{Fe}}^{2+}}$ **(kcal/mol)**			
	**Scheme I MP2/CBS**	**MP2/CBS-MP2/aug-cc-pVDZ**	**MP2/CBS-MP2/aug-cc-pVTZ**	**MP2/CBS-MP2/aug-cc-pVQZ**
Fe(H_2_O)	−602.8804	−6.23	−3.75	−1.12
Fe(H_2_O)_2_	−538.3929	−6.10	−3.74	−1.23
Fe(H_2_O)_4_	−442.5902	−6.05	−3.68	−1.35
Fe(H_2_O)_6_	−386.9860	−5.64	−3.44	−1.09
Fe(H_2_O)_3_(CH_3_O^−^)	−279.0221	−7.19	−4.11	−2.09
Fe(H_2_O)_3_(CH_3_S^−^)	−263.8079	−5.02	−2.56	−1.08
Fe(H_2_O)_3_(NH_2_CH_3_)	−425.3816	−6.66	−3.73	−1.39
Fe(H_2_O)_3_(HCOO^−^)	−307.3227	−6.54	−3.86	−1.76
Fe(H_2_O)_5_(CH_3_O^−^)	−252.6365	−6.56	−3.93	−1.97
Fe(H_2_O)_5_(CH_3_S^−^)	−241.5428	−4.96	−2.58	−1.07
Fe(H_2_O)_5_(NH_2_CH_3_)	−376.6431	−6.22	−3.53	−1.15
Fe(H_2_O)_5_(HCOO^−^)	−274.9742	−5.90	−3.60	−1.53
MSE	–	−6.09	−3.54	−1.40
MUE	–	6.09	3.54	1.40

Our results show that MP2/CBS energies obtained with scheme I are closer to those calculated with quadruple-zeta basis set than any of the other basis sets, as expected, and that the difference between the CBS and quadruple-zeta basis set is small, as can be confirmed by comparing the values for the mean signed error (MSE) and mean unsigned error (MUE) for each of them. We can also observe that the convergence of the reduction potential with the basis set is slow, since at quadruple-zeta the values are still 1.40 kcal/mol (in average) away from the CBS limit.

Using scheme II, developed by T. Helgaker and co-workers, to extrapolate $\Delta {\text{E}}_{\text{elec}}^{\text{F}{\text{e}}^{3+}/\text{F}{\text{e}}^{2+}}$ to the MP2/CBS level, we obtained the results shown in Table 4. This scheme uses the energies calculated with the aug-cc-pVTZ and aug-cc-pVQZ basis sets for the extrapolation. Since these basis sets are more complete that the ones used in scheme I, it might be expected that the reduction potentials extrapolated using scheme II are more accurate.

**Table 4:** $\Delta {\text{E}}_{\text{elec}}^{\text{F}{\text{e}}^{3+}/\text{F}{\text{e}}^{2+}}$obtained at the MP2/CBS level, extrapolated using scheme II for each model, and the differences between these energies and those obtained using scheme I and at the MP2/aug-cc-pVQZ level.

**Model**	$\Delta {\text{E}}_{\text{elec}}^{{\text{Fe}}^{3+}/{\text{Fe}}^{2+}}$ **(kcal/mol)**		
	**Scheme II MP2/CBS**	**Scheme I MP2/CBS – Scheme II MP2/CBS**	**Scheme II MP2/CBS - MP2/aug-cc-pVQZ**
Fe(H_2_O)	−602.8540	−0.0264	−1.0956
Fe(H_2_O)_2_	−538.2482	−0.1447	−1.0813
Fe(H_2_O)_4_	−442.1297	−0.4605	−0.8882
Fe(H_2_O)_6_	−386.5973	−0.3887	−0.7042
Fe(H_2_O)_3_(CH_3_O^−^)	−277.7082	−1.3139	−0.7751
Fe(H_2_O)_3_(CH_3_S^−^)	−263.7248	−0.0830	−0.9939
Fe(H_2_O)_3_(NH_2_CH_3_)	−424.9332	−0.4484	−0.9379
Fe(H_2_O)_3_(HCOO^−^)	−306.5217	−0.8010	−0.9611
Fe(H_2_O)_5_(CH_3_O^−^)	−251.2897	−1.3468	−0.6250
Fe(H_2_O)_5_(CH_3_S^−^)	−241.3777	−0.1651	−0.9084
Fe(H_2_O)_5_(NH_2_CH_3_)	−376.2997	−0.3434	−0.8075
Fe(H_2_O)_5_(HCOO^−^)	−274.2278	−0.7464	−0.7799
MSE	–	−0.52	−0.88
MUE	–	0.52	0.88

Besides the $\Delta {\text{E}}_{\text{elec}}^{\text{F}{\text{e}}^{3+}/\text{F}{\text{e}}^{2+}}$ extrapolated to the MP2/CBS level using scheme II, Table 4 also shows the difference between these values and those extrapolated using scheme I and calculated at the MP2/aug-cc-pVQZ level. From these results, we can observe that there is a slight difference between the scheme II MP2/CBS and MP2/aug-cc-pVQZ energies, with an average of 0.88 kcal/mol. This indicates that, for these systems, the values calculated with quadruple-zeta basis sets are not accurate enough, and extrapolation to the CBS limit is indeed necessary. Furthermore, it is interesting to note that the difference between both extrapolation schemes is small (MUE = 0.52 kcal/mol), but not insignificant, especially considering that both of these methods are expected to yield very accurate results. Although in some cases this difference is small enough that it can be disregarded [0.024 kcal/mol for Fe(H_2_O) and 0.0830 kcal/mol for Fe(H_2_O)_3_(CH_3_S^−^)], in cases such as that of Fe(H_2_O)_3_(CH_3_O^−^) and Fe(H_2_O)_5_(CH_3_O^−^) it is actually somewhat relevant (~ 1.3 kcal/mol). Since scheme II uses larger basis sets to perform the extrapolation, we chose those energies for the remainder of the work, particularly in the extrapolation to the CCSD(T)/CBS level. It is, however, worth mentioning that, even though the use of large basis sets to get more accurate results is a good practice in systems with a small number of atoms, such methods are impractical for large biological systems, in which case the system would have to be reduced significantly, introducing much larger errors.

Fortunately, DFT methods converge much faster than Post-HF methods in relation to the basis set completeness, and as such they will not be affected by these large basis set truncation errors, as seen here.

### Calculation of the Reduction Potential at the CCSD(T)/CBS Level

The last step in the calculation of the reference values for $\Delta {\text{E}}_{\text{elec}}^{\text{F}{\text{e}}^{3+}/\text{F}{\text{e}}^{2+}}$ is the extrapolation to the CCSD(T)/CBS level. After obtaining $\Delta {\text{E}}_{\text{elec}}^{\text{F}{\text{e}}^{3+}/\text{F}{\text{e}}^{2+}}$ at MP2/CBS, this last step can be easily done by adding to this value the difference between the reduction potentials calculated with MP2 and CCSD(T) (*E*_*CCSD*(*T*)−*MP*2_) at the same basis set, aug-cc-pVDZ in our case. This widely used approximation is based on the fact that the difference in correlation energy between CCSD(T) and MP2 does not depend strongly on the basis set, in particular for medium/large basis sets. Due to the high computational cost of CCSD(T) calculations, we obtained $\Delta {\text{E}}_{\text{elec}}^{\text{F}{\text{e}}^{3+}/\text{F}{\text{e}}^{2+}}$ with this method with the aug-cc-pVDZ basis set. Previous studies have shown that using a larger basis set in this correction does not significantly alter the extrapolated value, and that the largest difference between using double or triple-zeta when obtaining *E*_*CCSD*(*T*)−*MP*2_ was < 0.1 kcal/mol (Jurečka and Hobza, 2002). Jurečka et al. (2006) have also previously stated that double-zeta basis sets are complete enough to obtain a good extrapolation to the CBS limit, and using larger basis sets requires more computational time without much benefit.

Table 5 presents the results $\Delta {\text{E}}_{\text{elec}}^{\text{F}{\text{e}}^{3+}/\text{F}{\text{e}}^{2+}}$ at the CCSD(T)/CBS level (as stated previously, CCSD(T)/CBS was extrapolated using the results obtained with extrapolation scheme II). We can observe that the difference between both CCSD(T) and MP2 at the CBS limit is significant, which is a result of the relevant role that correlation energy has in the case of our chemical transformation, which is expectable as an electron is being taken out from the system.

**Table 5:** $\Delta {\text{E}}_{\text{elec}}^{\text{F}{\text{e}}^{3+}/\text{F}{\text{e}}^{2+}}$ obtained at the CCSD(T)/CBS level and at the MP2/CBS level (extrapolated using scheme II), as well as the difference between these two values for each model.

**Model**	$\Delta {\text{E}}_{\text{elec}}^{\text{F}{\text{e}}^{3+}/\text{F}{\text{e}}^{2+}}$ **(kcal/mol)**		
	**CCSD(T)/CBS**	**MP2/CBS**	**CCSD(T)/CBS - MP2/CBS**
Fe(H_2_O)	−593.8053	−602.8540	9.05
Fe(H_2_O)_2_	−532.2488	−538.2482	6.00
Fe(H_2_O)_4_	−437.8827	−442.1297	4.25
Fe(H_2_O)_6_	−383.3053	−386.5973	3.29
Fe(H_2_O)_3_(CH_3_O^−^)	−268.8103	−277.7082	8.90
Fe(H_2_O)_3_(CH_3_S^−^)	−263.1074	−263.7248	0.62
Fe(H_2_O)_3_(NH_2_CH_3_)	−419.8000	−424.9332	5.13
Fe(H_2_O)_3_(HCOO^−^)	−302.7982	−306.5217	3.72
Fe(H_2_O)_5_(CH_3_O^−^)	−242.1318	−251.2897	9.16
Fe(H_2_O)_5_(CH_3_S^−^)	−239.3788	−241.3777	2.00
Fe(H_2_O)_5_(NH_2_CH_3_)	−372.7251	−376.2997	3.57
Fe(H_2_O)_5_(HCOO^−^)	−268.0456	−274.2278	6.18

The energies extrapolated to the MP2/CBS level are much closer to the true value than those calculated using a truncated basis set, but these are still, nonetheless, approximations with associated errors that are introduced at different points throughout our calculations. Furthermore, the conversion of the MP2 level into the CCSD(T) level at the CBS limit is also the cause of small error. Overall, it must be taken into account that these results were obtained through several approximations, each of which adding a small error to our final values. In total, we expect our reference values ($\Delta {\text{E}}_{\text{elec}}^{\text{F}{\text{e}}^{3+}/\text{F}{\text{e}}^{2+}}$ calculated at the CCSD(T)/CBS level) to have an associated uncertainty of 1 kcal/mol or less, in most of the cases. As such, a qualitative distinction between density functionals having a difference smaller than 1 kcal/mol between them should not be made.

### Benchmarking of the DFT Functionals

After obtaining the reference values for $\Delta {\text{E}}_{\text{elec}}^{\text{F}{\text{e}}^{3+}/\text{F}{\text{e}}^{2+}}$ of iron in each of the complexes, we can now use these results to evaluate the performance of the 44 density functionals we have selected. It should be noted that this benchmark study does not represent the overall quality of each of the DFT, but merely indicates, for these systems in specific (which we expect to be representative of aminoacid side-chain coordination shells), which of them are better at estimating the chemical property we wish to study (iron reduction potential, in our case).

From the optimized geometries for all systems, we performed single point calculation with each of the 44 density functionals, and calculated $\Delta {\text{E}}_{\text{elec}}^{\text{F}{\text{e}}^{3+}/\text{F}{\text{e}}^{2+}}$ using the basis set 6-311+G(2df,2p). We selected a large basis set in order to minimize truncation errors, which means that the fluctuations on the results arise essentially from the density functional. The values obtained for $\Delta {\text{E}}_{\text{elec}}^{\text{F}{\text{e}}^{3+}/\text{F}{\text{e}}^{2+}}$ will be compared to the reference values calculated at the CCSD(T)/CBS level.

In order to make discussing the results easier, and because our study involves several different systems, we decided to group our models in 3 distinct groups (Figure 1). Group A comprises the iron complexes with only water as their ligand, with coordination numbers 1, 2, 4, and 6, whereas groups B and C include complexes which contain one ligand that represents the side chain of an amino acid. What differentiates these 2 groups is the coordination number of the iron ion: 4 for group B and 6 for group C. The iron is complexed with 3 and 5 water molecules, respectively, plus the ligand simulating the amino acid side chain.

For the next part of this paper, we will only analyze the ten functionals that gave the best performance for each case. We decided to present our results in this fashion in order to make a more focused and easy-to-read discussion. A full table displaying the results for all the DFs tested is available in the Supporting Information section of this paper.

We begin our discussion with the systems belonging to group A.

Table 6 represents the results obtained for the ten density functionals that showed the best performance for this group. Performance is measured as the difference between $\Delta {\text{E}}_{\text{elec}}^{\text{F}{\text{e}}^{3+}/\text{F}{\text{e}}^{2+}}$ calculated with each DFT and the reference value (MUE). We also show the maximum error (MaxE), which is the model were the difference between the value calculated with the reference value and the energy calculated with the density functional was higher.

**Table 6:** Top ten best performing functionals for group A complexes.

	**Functional**	**Type**	**%HF_**exchange**_**	**MUE**	**MaxE**	**Fe(H_**2**_O)**	**Fe(H_**2**_O)_**2**_**	**Fe(H_**2**_O)_**4**_**	**Fe(H_**2**_O)_**6**_**
1	BB1K	hm-GGA	42.00	0.67	1.76	−0.47	0.21	−0.26	1.76
2	mPWB1K	hm-GGA	44.00	0.99	1.93	0.44	1.28	0.29	1.93
3	mPW1N	h-GGA	40.60	2.23	3.49	1.21	2.55	1.68	3.49
4	BMK	hm-GGA	42.00	2.41	3.62	3.39	1.52	−1.10	3.62
5	mPW1B95	hm-GGA	31.00	2.49	4.12	−2.39	−4.12	−2.56	0.89
6	M06-2X	hm-GGA	54.00	2.53	4.50	4.50	3.79	0.89	0.93
7	mPW1K	h-GGA	42.80	2.62	3.51	1.70	3.30	1.98	3.51
8	MN12-SX	hm-NGA	25.00/–	2.98	7.43	0.81	−1.43	2.25	7.43
9	B3LYP	h-GGA	20.00	3.03	5.41	−0.67	−5.41	−2.61	3.42
10	mPW2PLYP	hh-GGA	55.00	3.08	4.69	−3.55	−1.89	−4.69	−2.19

*For each functional, we highlighted (red) the model with the largest difference between reference and calculated value*.

Taking these results into account, we can further divide the DFs into 3 separate groups. Group I includes all the density functionals with MUE between 0 and 2.31 kcal/mol (which corresponds to an error below 0.1 V per electron transfer). Group II comprises functionals with MUE between 2.31 and 4.62 kcal/mol (between 0.1 and 0.2 V per electron transfer) and group III encompasses those whose MUE is higher than 4.62 kcal/mol. The density functionals whose performance puts them in group I are BB1K, mPWB1K, and mPW1N. It is interesting to note that these DFs all belong to either the hybrid-meta-GGA or hybrid-GGA class, and that in the whole table only two functionals do not belong to these classes. The first four functionals all have a rather high HF exchange percentage, higher than 40%. The very popular B3LYP functional appears at 9th place in our table as well, and its MUE puts it in group II. It is also worth noting that the Fe(H_2_O)_6_ system is the one where the largest error is more frequently found.

We could also measure the performance of the tested functionals in terms of MaxE. However, and taking the results in Table 6 into account, we can observe that both MUE and MaxE show a similar trend, and that those functionals with a smaller MUE also tend to have a smaller MaxE, with a few exceptions. For this reason, we decided to continue to rank the DFs in terms of MUE.

For group B, we performed the same analysis as group A. As stated previously, this group consists of systems with three water molecules and one ligand coordinated to the iron ion in a tetrahedral geometry. Each ligand represents a different amino acid side chain (CH_3_O^−^ for serine, HCOO^−^ for glutamate and aspartate, CH_3_S^−^ for cysteine and NH_2_CH_3_ for lysine), which correspond to the most common residues that participate in iron complexes of proteins.

The results for group B (Table 7), show that the DFs that display a better accuracy for this group are mostly similar to those that performed better for system A as well. The density functionals that belong to group I in this case are BMK and B3LYP, which are, yet again, either hybrid-meta-GGAs or hybrid-GGAs. The HF correlation percentage for most of the top ten functionals is also higher than 40%, with MN-12L as a remarkable exception. The system that contributes the most to the maximum error in this group is Fe(CH_3_S^−^)(H_2_O)_3_.

**Table 7:** Top ten best performing functionals for group B complexes.

	**Functional**	**Type**	**%HF_**exchange**_**	**MUE**	**MaxE**	**Fe(H_**2**_O)_**3**_ (CH_**3**_O^**−**^)**	**Fe(H_**2**_O)_**3**_ (CH_**3**_S^**−**^)**	**Fe(H_**2**_O)_**3**_ (NH_**2**_CH_**3**_)**	**Fe(H_**2**_O)_**3**_ (HCOO^**−**^)**
1	BMK	hm-GGA	42.00	2.02	3.71	3.71	−1.24	1.84	−1.30
2	B3LYP	h-GGA	20.00	2.10	6.22	0.02	−0.63	−1.55	−6.22
3	wB97X-D	h-GGA	22.20/100.00	2.33	4.21	−4.21	−0.82	0.38	−3.90
4	BB1K	hm-GGA	42.00	2.44	4.33	1.59	−4.33	0.93	−2.92
5	mPWB1K	hm-GGA	44.00	2.53	3.97	2.61	−3.97	1.61	−1.93
6	mPW1N	h-GGA	40.60	2.65	4.04	3.68	−4.04	2.59	−0.27
7	mPW1B95	hm-GGA	31.00	2.72	6.02	−0.69	−2.45	−1.71	−6.02
8	mPW1K	hm-GGA	44.00	2.96	4.30	4.22	−4.30	3.01	0.30
9	MN12-L	m-NGA	–	2.98	4.50	4.03	4.50	0.56	−2.84
10	M06-2X	hm-GGA	54.00	3.07	6.93	6.93	−2.52	2.21	0.63

*For each functional, we highlighted (red) the model with the largest difference between reference and calculated value*.

And finally, we reach our last group, group C, composed of iron complexes with coordination number 6, in which one of the ligand is a chemical group representing an amino acid side chain, and the other five are water molecules. The results obtained for the benchmarking performed for group C are present in Table 8. Six functionals have MUE values < 2.31 kcal/mol, placing them in group I. These are mPW1B95, PBE1PBE, B3PW91, wB97X-D, BB1K, and mPWB1K. The percentage of HF exchange is not as high for this group, but still some of the functionals placed in the top ten have more the 40% HF exchange. Again for group C, all of the functionals in group I, and indeed in the whole table, are either hybrid-meta-GGAs or hybrid-GGAs. In accordance with the results for group B, for group C it is also the complex with CH_3_S^−^ that contributed the most times to the MaxE. B3LYP shows a good performance in this group as well, ranking in the 7th position. We also assessed the contribution of the dispersion effect on the iron complexes of group C, which are those that have a higher number of ligands and, therefore, where the dispersion interactions should be more noticeable. In this sense, we calculated the Grimme's dispersion correction (Grimme et al., 2010; Goerigk and Grimme, 2011) with the top ten functionals (Table 8) and concluded that its inclusion marginally enhances the results, as the average MUE was reduced by ~0.15 kcal/mol. However, considering the precision of the reference CCSD(T)/CBS method (about 0.5–1 kcal/mol), we can assume that the inclusion of the dispersion correction does not significantly influence the results and, consequently, the gathered illations.

**Table 8:** Top ten best performing functionals for group C complexes.

	**Functional**	**Type**	**%HF_**exchange**_**	**MUE**	**MaxE**	**Fe(H_**2**_O)_**5**_ (CH_**3**_O^**−**^)**	**Fe(H_**2**_O)_**5**_ (CH_**3**_S^**−**^)**	**Fe(H_**2**_O)_**5**_ (NH_**2**_CH_**3**_)**	**Fe(H_**2**_O)_**5**_ (HCOO^**−**^)**
1	mPW1B95	hm-GGA	31.00	1.65	3.26	−1.73	−3.26	0.86	0.74
2	PBE1PBE	h-GGA	25.00	1.71	3.48	−2.51	−3.48	−0.61	−0.25
3	B3PW91	h-GGA	20.00	1.73	2.60	−2.06	−2.60	1.23	1.02
4	wB97X-D	h-GGA	22.20/100.00	1.99	3.43	0.38	0.77	3.43	3.39
5	BB1K	hm-GGA	42.00	2.03	4.47	0.23	−4.47	1.68	1.76
6	mPWB1K	hm-GGA	44.00	2.29	3.98	1.05	−3.98	1.92	2.20
7	B3LYP	h-GGA	20.00	2.38	3.22	−0.41	−3.08	3.22	2.82
8	B1LYP	h-GGA	25.00	2.81	7.23	−2.53	−7.23	0.90	0.57
9	B97-1	h-GGA	21.00	2.82	4.10	−3.38	−4.10	−0.35	−3.46
10	M06-2X	hm-GGA	54.00	2.91	4.77	4.77	−2.43	1.39	3.05

*For each functional, we highlighted (red) the model with the largest difference between reference and calculated value*.

Having analyzed the performance of the 44 density functionals in estimating the reduction potentials of iron complexes in the three separate groups, we will now make an overall appreciation of their performance for all the systems studies as a whole.

Table 9 presents the ten functionals that showed the best performance in calculating $\Delta {\text{E}}_{\text{elec}}^{\text{F}{\text{e}}^{3+}/\text{F}{\text{e}}^{2+}}$ for the studied iron complexes. Three functionals MUE values that put them in group I: BB1K, mPWB1K, and mPW1B95. All of them belong to the hybrid-meta-GGA class of functionals. The very popular B3LYP functional ranks 5th in this overall analysis. All functionals present in the top ten are either hybrid-meta-GGAs or hybrid-GGAs, and the percentage of HF exchange is higher than 40% in most of the cases. We can therefore conclude that both of these classes are the best at estimating the $\Delta {\text{E}}_{\text{elec}}^{\text{F}{\text{e}}^{3+}/\text{F}{\text{e}}^{2+}}$ of the iron complexes studied, and should be used when calculating the reduction potential of iron complexes.

**Table 9:** Top ten best performing functionals for all the complexes studied.

	**Functional**	**Type**	**%HF_**exchange**_**	**MUE**	**MaxE**	**MaxE Complex**
1	BB1K	hm-GGA	42.00	1.72	4.47	Fe(H_2_O)_5_(CH_3_S^−^)
2	mPWB1K	hm-GGA	44.00	1.93	3.98	Fe(H_2_O)_5_(CH_3_S^−^)
3	mPW1B95	hm-GGA	31.00	2.28	6.02	Fe(H_2_O)_3_(HCOO^−^)
4	BMK	hm-GGA	42.00	2.49	3.93	Fe(H_2_O)_5_(HCOO^−^)
5	B3LYP	h-GGA	20.00	2.51	6.22	Fe(H_2_O)_3_(HCOO^−^)
6	B3PW91	h-GGA	20.00	2.72	7.23	Fe(H_2_O)_3_(HCOO^−^)
7	mPW1N	h-GGA	40.60	2.74	4.04	Fe(H_2_O)_3_(CH_3_S^−^)
8	M06-2X	hm-GGA	54.00	2.84	6.93	Fe(H_2_O)_3_(CH_3_O^−^)
9	mPW1K	h-GGA	42.80	3.03	4.30	Fe(H_2_O)_3_(CH_3_S^−^)
10	PBE1PBE	h-GGA	25.00	3.08	7.21	Fe(H_2_O)_3_(HCOO^−^)

*The complex to which the MaxE corresponds to is also shown*.

## Conclusion

In our study, we analyzed the performance of 44 density functionals in estimating the electronic component of the reduction potential at 0 K of 16 different iron complexes, with a special interest in iron complexes with ligands similar to some amino acid side-chains. We were able to conclude which of these DFs gave results closer to reference values calculated using very high level computational methods, with posterior extrapolation to the very accurate CCSD(T)/CBS level of theory, which was done with two different extrapolation schemes. An initial geometry optimization for each of the 16 systems was carried out with MP2/6-311+G(d,p) level of theory, followed by single-point calculations with each of the 44 selected functional with the 6-311+G(2df,2p) basis set. From the difference between the energies obtained with Fe^2+^ and Fe^3+^, we calculated $\Delta {\text{E}}_{\text{elec}}^{\text{F}{\text{e}}^{3+}/\text{F}{\text{e}}^{2+}}$ for all complexes, and then compared the results obtained with the reference values, ranking the DFs according to this difference.

Our results show that the difference between the extrapolated values for the level MP2/CBS calculated with both extrapolation schemes is very small (average difference is 0.52 kcal/mol). For the final extrapolation to the CCSD(T)/CBS level of the reduction potentials we selected the values calculated with scheme II, since it uses results obtained with a larger basis set for the extrapolation.

The benchmarking study showed us that the best functionals to calculate the reduction potential of iron complexes, especially those associated to biological systems, belong to the hybrid or hybrid-meta-GGAs classes, and have a high percentage of HF exchange. Overall, BB1K, mPWB1K, and mPW1B95 were the functionals that performed better for all iron complexes studied (average MUE 1.72, 1.93 and 2.28 kcal/mol, respectively). The very popular functional B3LYP gave a rather good performance, ranking 5th in our list of best functionals.

As a final remark it is important to note that there is an estimate of a 1 kcal/mol error in our reference values, which means that it is very difficult to assess the overall performance of functionals that differ by <1 kcal/mol between them. Nevertheless, our results are significant to show which DFs are more likely to perform well with iron complexes that incorporate biological systems, even if we are not able to show which, among all of them, is the single best density functional.

## Data Availability

All datasets generated for this study are included in the manuscript and/or the Supplementary Files.

## Author Contributions

RL performed most of the calculations, analyzed the results, and contributed to the writing of the manuscript. DG and PP performed a part of the calculations, analyzed the results, and contributed to the writing of the manuscript. MR and PF planned the research, analyzed the results, and contributed to the writing of the manuscript.

### Conflict of Interest Statement

The authors declare that the research was conducted in the absence of any commercial or financial relationships that could be construed as a potential conflict of interest.
