# Supplementary material for: Benchmark of Density Functionals for the Calculation of the Redox Potential of Fe3+/Fe2+ Within Protein Coordination Shells
Source: Front Chem. 2019 Jun 5;7:391. doi: 10.3389/fchem.2019.00391 (PMC6560050; doi:10.3389/fchem.2019.00391)
Supplement: Supplementary file 1 [file Data_Sheet_1.pdf]

Benchmark of Density Functionals for the Calculation of the Redox potential of Fe^{3+}/Fe^{2+} within protein coordination shells.

Risnita Vicky Listyarini[1], Diana Sofia Gesto[2], Pedro Paiva, Maria João Ramos and Pedro Alexandrino Fernandes*
UCIBIO@REQUIMTE, Departamento de Química e Bioquímica, Faculdade de Ciências Universidade do Porto, Rua do Campo Alegre s/n,4169-007 Porto, Portugal
[1]present address: Chemistry Education Study Program, Sanata Dharma University, Indonesia
[2]present address: UCIBIO, Departamento de Química, Faculdade de Ciências e Tecnologia, Universidade Nova de Lisboa, Caparica, Portugal.
*e-mail: pafernan@fc.up.pt

SUPPORTING INFORMATION

Single point energies for the calculation of the reference values:

Table 1 – Single point energies for the Fe^{2+} complexes calculated with MP2/aug-cc-pVXZ (X = 2, 3 and 4) and CCSD(T)/aug-cc-pVDZ levels of theory.

Model	$E_{elec}^{Fe^{2+}}$ (Hartree)			
	MP2/ aug-cc-pVDZ	MP2/ aug-cc-pVTZ	MP2/ aug-cc-pVQZ	CCSD(T)/ aug-cc-pVDZ
$Fe(H_2O)$	-1338.1526	-1338.2506	-1338.2864	-1338.1723
$Fe(H_2O)_2$	-1414.5291	-1414.6952	-1414.7543	-1414.5631
$Fe(H_2O)_4$	-1567.2165	-1567.5181	-1567.6234	-1567.2782
$Fe(H_2O)_6$	-1719.8361	-1720.2703	-1720.4207	-1719.9245
$Fe(H_2O)_3(CH_3O^-)$	-1606.1520	-1606.4912	-1606.6076	-1606.2350
$Fe(H_2O)_3(CH_3S^-)$	-1928.7674	-1929.0934	-1929.2054	-1928.8622
$Fe(H_2O)_3(NH_2CH_3)$	-1586.5599	-1586.8856	-1586.9963	-1586.6492
$Fe(H_2O)_3(HCOO^-)$	-1680.0786	-1680.4710	-1680.6068	-1680.1652
$Fe(H_2O)_5(CH_3O^-)$	-1758.7365	-1759.2086	-1759.3698	-1758.8455
$Fe(H_2O)_5(CH_3S^-)$	-2081.3470	-2081.8056	-2081.9624	-2081.4662
$Fe(H_2O)_5(NH_2CH_3)$	-1739.1688	-1739.6271	-1739.7827	-1739.2847
$Fe(H_2O)_5(HCOO^-)$	-1832.6733	-1833.1981	-1833.3789	-1832.7765

Table 2 - Single point energies for the Fe^{2+} complexes calculated with MP2/aug-cc-pVXZ (X = 2, 3 and 4) and CCSD(T)/aug-cc-pVDZ levels of theory.

**Model**	$E_{elec}^{Fe^{3+}}$ **(Hartree)**			
	**MP2/ aug-cc-pVDZ**	**MP2/ aug-cc-pVTZ**	**MP2/ aug-cc-pVQZ**	**CCSD(T)/ aug-cc-pVDZ**
**$Fe(H_2O)$**	-1337.2017	-1337.2958	-1337.3274	-1337.2359
**$Fe(H_2O)_2$**	-1413.6809	-1413.8432	-1413.8982	-1413.7244
**$Fe(H_2O)_4$**	-1566.5208	-1566.8186	-1566.9203	-1566.5893
**$Fe(H_2O)_6$**	-1719.2284	-1719.6590	-1719.8057	-1719.3220
**$Fe(H_2O)_3(CH_3O^-)$**	-1605.7188	-1606.0531	-1606.1662	-1605.8160
**$Fe(H_2O)_3(CH_3S^-)$**	-1928.3550	-1928.6770	-1928.7867	-1928.4508
**$Fe(H_2O)_3(NH_2CH_3)$**	-1585.8926	-1586.2137	-1586.3206	-1585.9901
**$Fe(H_2O)_3(HCOO^-)$**	-1679.5993	-1679.9874	-1680.1199	-1679.6918
**$Fe(H_2O)_5(CH_3O^-)$**	-1758.3444	-1758.8122	-1758.9703	-1758.4680
**$Fe(H_2O)_5(CH_3S^-)$**	-2080.9700	-2081.4248	-2081.5792	-2081.0924
**$Fe(H_2O)_5(NH_2CH_3)$**	-1738.5785	-1739.0325	-1739.1843	-1738.7001
**$Fe(H_2O)_5(HCOO^-)$**	-1832.2445	-1832.7657	-1832.9432	-1832.3575

Benchmarking of DFT

Group A

Table 3 – Benchmarking of the density functionals for the complexes of group A.

**DF**	**$Fe(H_2O)$**			
	$E_{elec}^{Fe^{2+}}$ **(Hartree)**	$E_{elec}^{Fe^{3+}}$ **(Hartree)**	$\Delta E_{elec}^{Fe^{3+}/Fe^{2+}}$ **(kcal/mol)**	**CCSD(T)/CBS - DF**
**BB1K**	-1339.4641	-1338.5186	-593.3347	-0.47
**mPWB1K**	-1339.4700	-1338.5230	-594.2446	0.44
**mPW1N**	-1339.3792	-1338.4310	-595.0196	1.21
**BMK**	-1338.7597	-1337.8080	-597.1939	3.39
**mPW1B95**	-1339.4929	-1338.5504	-591.4158	-2.39
**M06-2X**	-1339.2761	-1338.3226	-598.3052	4.50
**mPW1K**	-1339.3756	-1338.4266	-595.5021	1.70
**MN12-SX**	-1339.1635	-1338.2159	-594.6180	0.81
**B3LYP**	-1339.3599	-1338.4147	-593.1370	-0.67
**mPW2PLYP**	-1338.9143	-1337.9737	-590.2511	-3.55
**B3PW91**	-1339.2857	-1338.3431	-591.5199	-2.29
**PBE1PBE**	-1339.0294	-1338.0892	-589.9775	-3.83
**M11-L**	-1339.4149	-1338.4672	-594.6719	0.87
**B2PLYP**	-1338.8809	-1337.9422	-589.0218	-4.78
**OVWN5**	-1340.9218	-1339.9738	-594.8527	1.05
**OPL**	-1340.8979	-1339.9485	-595.7362	1.93
**B1LYP**	-1339.3199	-1338.3797	-589.9819	-3.82

**MN12-L**	-1338.9787	-1338.0383	-590.1106	-3.69
**M05-2X**	-1339.2656	-1338.3076	-601.1240	7.32
**B2GPPLYP**	-1338.7331	-1337.7944	-589.0620	-4.74
**wB97X-D**	-1339.3340	-1338.3854	-595.2348	1.43
**DSD-BLYP**	-1338.6100	-1337.6732	-587.8628	-5.94
**TPSSh**	-1339.3460	-1338.4121	-586.0073	-7.80
**SVWN**	-1336.9992	-1336.0401	-601.8745	8.07
**BHandH**	-1337.0821	-1336.1434	-589.0212	-4.78
**M11**	-1339.2180	-1338.2583	-602.1901	8.38
**N12**	-1339.7205	-1338.7763	-592.4643	-1.34
**HCTH407**	-1339.8546	-1338.9123	-591.3041	-2.50
**BP86**	-1339.5016	-1338.5624	-589.3538	-4.45
**M06**	-1339.2266	-1338.2953	-584.4153	-9.39
**B97-1**	-1339.1984	-1338.2764	-578.5700	-15.24
**B3P86**	-1340.0483	-1339.0832	-605.5856	11.78
**B97-2**	-1339.5362	-1338.6162	-577.2931	-16.51
**M05**	-1339.3076	-1338.3798	-582.2297	-11.58
**B97-D3**	-1339.7673	-1338.8311	-587.4587	-6.35
**BPW91**	-1339.4446	-1338.5097	-586.6806	-7.12
**mPWB95**	-1339.5642	-1338.6260	-588.7169	-5.09
**BPBE**	-1339.3222	-1338.3886	-585.8448	-7.96
**OLYP**	-1339.6082	-1338.6776	-583.9829	-9.82
**G96LYP**	-1339.4746	-1338.5420	-585.2298	-8.58
**VSXC**	-1339.8472	-1338.9258	-578.2130	-15.59
**OTPSS**	-1339.5611	-1338.6363	-580.2995	-13.51
**M06-L**	-1339.2699	-1338.3516	-576.2589	-17.55
**SPW91**	-1335.0592	-1334.1444	-574.0564	-19.75

Table 3 (cont.)

**DF**	**$Fe(H_2O)_2$**			
	**$E_{elec}^{Fe^{2+}}$ (Hartree)**	**$E_{elec}^{Fe^{3+}}$ (Hartree)**	**$\Delta E_{elec}^{Fe^{3+}/Fe^{2+}}$ (kcal/mol)**	**CCSD(T)/CBS - DF**
**BB1K**	-1416.0029	-1415.1544	-532.4575	0.21
**mPWB1K**	-1416.0080	-1415.1578	-533.5318	1.28
**mPW1N**	-1415.9278	-1415.0756	-534.7981	2.55
**BMK**	-1415.2997	-1414.4490	-533.7690	1.52
**mPW1B95**	-1416.0382	-1415.1966	-528.1333	-4.12
**M06-2X**	-1415.8236	-1414.9694	-536.0400	3.79
**mPW1K**	-1415.9230	-1415.0696	-535.5442	3.30
**MN12-SX**	-1415.6768	-1414.8309	-530.8228	-1.43
**B3LYP**	-1415.9419	-1415.1023	-526.8369	-5.41
**mPW2PLYP**	-1415.3591	-1414.5139	-530.3597	-1.89
**B3PW91**	-1415.8360	-1414.9978	-526.0004	-6.25
**PBE1PBE**	-1415.5254	-1414.6872	-525.9879	-6.26
**M11-L**	-1415.9626	-1415.1228	-526.9542	-5.29
**B2PLYP**	-1415.3193	-1414.4759	-529.2421	-3.01
**OVWN5**	-1417.7947	-1416.9523	-528.6071	-3.64
**OPL**	-1417.7665	-1416.9231	-529.2271	-3.02

**B1LYP**	-1415.8692	-1415.0317	-525.5398	-6.71
**MN12-L**	-1415.4721	-1414.6362	-524.5226	-7.73
**M05-2X**	-1415.8318	-1414.9742	-538.1597	5.91
**B2GPPLYP**	-1415.1340	-1414.2925	-528.0543	-4.19
**wB97X-D**	-1415.8578	-1415.0398	-513.2952	-18.95
**DSD-BLYP**	-1414.9804	-1414.1423	-525.8781	-6.37
**TPSSh**	-1415.9203	-1415.0927	-519.3212	-12.93
**SVWN**	-1413.2376	-1412.3984	-526.5652	-5.68
**BHandH**	-1413.1971	-1412.3575	-526.8570	-5.39
**M11**	-1415.7690	-1414.9077	-540.4865	8.24
**N12**	-1416.2573	-1415.4331	-517.2209	-15.03
**HCTH407**	-1416.4264	-1415.5946	-521.9637	-10.29
**BP86**	-1416.0866	-1415.2642	-516.0312	-16.22
**M06**	-1415.7669	-1414.9399	-518.9980	-13.25
**B97-1**	-1415.7482	-1414.9341	-510.8586	-21.39
**B3P86**	-1416.8078	-1415.9478	-539.6412	7.39
**B97-2**	-1416.0803	-1415.2681	-509.6889	-22.56
**M05**	-1415.8449	-1415.0219	-516.4510	-15.80
**B97-D3**	-1416.3114	-1415.4850	-518.5488	-13.70
**BPW91**	-1416.0176	-1415.1989	-513.7508	-18.50
**mPWB95**	-1416.1320	-1415.3104	-515.5580	-16.69
**BPBE**	-1415.8709	-1415.0533	-513.0091	-19.24
**OLYP**	-1416.1606	-1415.3419	-513.7935	-18.46
**G96LYP**	-1416.0380	-1415.2229	-511.5200	-20.73
**VSXC**	-1416.4459	-1415.6326	-510.3328	-21.92
**OTPSS**	-1416.0953	-1415.2841	-509.0602	-23.19
**M06-L**	-1415.8323	-1415.0241	-507.1218	-25.13
**SPW91**	-1410.7951	-1409.9996	-499.2215	-33.03

Table 3 (cont.)

**DF**	**$Fe(H_2O)_4$**			
	**$E_{elec}^{Fe^{2+}}$ (Hartree)**	**$E_{elec}^{Fe^{3+}}$ (Hartree)**	**$\Delta E_{elec}^{Fe^{3+}/Fe^{2+}}$ (kcal/mol)**	**CCSD(T)/CBS - DF**
**BB1K**	-1569.0091	-1568.3117	-437.6232	-0.26
**mPWB1K**	-1569.0132	-1568.3150	-438.1736	0.29
**mPW1N**	-1568.9532	-1568.2527	-439.5591	1.68
**BMK**	-1568.3047	-1567.6086	-436.7861	-1.10
**mPW1B95**	-1569.0544	-1568.3607	-435.3240	-2.56
**M06-2X**	-1568.8537	-1568.1544	-438.7772	0.89
**mPW1K**	-1568.9467	-1568.2457	-439.8672	1.98
**MN12-SX**	-1568.6410	-1567.9396	-440.1314	2.25
**B3LYP**	-1569.0281	-1568.3344	-435.2726	-2.61
**mPW2PLYP**	-1568.1816	-1567.4913	-433.1924	-4.69
**B3PW91**	-1568.8585	-1568.1660	-434.5372	-3.35
**PBE1PBE**	-1568.4411	-1567.7502	-433.5413	-4.34
**M11-L**	-1568.9912	-1568.2912	-439.2623	1.38
**B2PLYP**	-1568.1288	-1567.4395	-432.5153	-5.37
**OVWN5**	-1571.4407	-1570.7455	-436.2572	-1.63

**OPL**	-1571.4038	-1570.7078	-436.7755	-1.11
**B1LYP**	-1568.8919	-1568.2011	-433.4836	-4.40
**MN12-L**	-1568.3939	-1567.6964	-437.6490	-0.23
**M05-2X**	-1568.9014	-1568.1995	-440.4075	2.52
**B2GPPLYP**	-1567.8716	-1567.1860	-430.1665	-7.72
**wB97X-D**	-1568.9228	-1568.2264	-437.0164	-0.87
**DSD-BLYP**	-1567.6584	-1566.9765	-427.8699	-10.01
**TPSSh**	-1568.9895	-1568.3051	-429.4982	-8.38
**SVWN**	-1565.6131	-1564.9324	-427.1137	-10.77
**BHandH**	-1565.3513	-1564.6714	-426.6512	-11.23
**M11**	-1568.8049	-1568.0940	-446.0419	8.16
**N12**	-1569.2475	-1568.5702	-425.0191	-12.86
**HCTH407**	-1569.4781	-1568.8000	-425.5054	-12.38
**BP86**	-1569.1704	-1568.4936	-424.7380	-13.14
**M06**	-1568.7845	-1568.1055	-426.0890	-11.79
**B97-1**	-1568.8047	-1568.1164	-431.9229	-5.96
**B3P86**	-1570.2483	-1569.5350	-447.5887	9.71
**B97-2**	-1569.1298	-1568.4418	-431.6776	-6.21
**M05**	-1568.8561	-1568.1796	-424.5095	-13.37
**B97-D3**	-1569.3133	-1568.6427	-420.8323	-17.05
**BPW91**	-1569.0782	-1568.4039	-423.1692	-14.71
**mPWB95**	-1569.1723	-1568.5021	-420.5820	-17.30
**BPBE**	-1568.8828	-1568.2096	-422.4507	-15.43
**OLYP**	-1569.1733	-1568.5039	-420.0593	-17.82
**G96LYP**	-1569.0789	-1568.4086	-420.6378	-17.24
**VSXC**	-1569.5753	-1568.9035	-421.5678	-16.31
**OTPSS**	-1569.0875	-1568.4166	-421.0143	-16.87
**M06-L**	-1568.8930	-1568.2291	-416.6017	-21.28
**SPW91**	-1562.1678	-1561.5288	-401.0219	-36.86

Table 3 (cont.)

**DF**	**$Fe(H_2O)_6$**				**Group A**	
	**$E_{elec}^{Fe^{2+}}$ (Hartree)**	**$E_{elec}^{Fe^{3+}}$ (Hartree)**	**$\Delta E_{elec}^{Fe^{3+}/Fe^{2+}}$ (kcal/mol)**	**CCSD(T)/CBS - DF**	**MUE**	**MaxE**
**BB1K**	-1721.9477	-1721.3341	-385.0649	1.76	0.67	1.76
**mPWB1K**	-1721.9517	-1721.3377	-385.2394	1.93	0.99	1.93
**mPW1N**	-1721.9091	-1721.2927	-386.7925	3.49	2.23	3.49
**BMK**	-1721.2418	-1720.6252	-386.9211	3.62	2.41	3.62
**mPW1B95**	-1722.0021	-1721.3899	-384.1983	0.89	2.49	4.12
**M06-2X**	-1721.8115	-1721.1991	-384.2310	0.93	2.53	4.50
**mPW1K**	-1721.9011	-1721.2847	-386.8176	3.51	2.62	3.51
**MN12-SX**	-1721.5426	-1720.9199	-390.7376	7.43	2.98	7.43
**B3LYP**	-1722.0406	-1721.4243	-386.7272	3.42	3.03	5.41
**mPW2PLYP**	-1720.9366	-1720.3292	-381.1135	-2.19	3.08	4.69
**B3PW91**	-1721.8077	-1721.1946	-384.7135	1.41	3.32	6.25
**PBE1PBE**	-1721.2857	-1720.6755	-382.9107	-0.39	3.71	6.26
**M11-L**	-1721.9599	-1721.3369	-390.9792	7.67	3.80	7.67
**B2PLYP**	-1720.8701	-1720.2632	-380.8292	-2.48	3.91	5.37

**OVWN5**	-1725.0188	-1724.3929	-392.7325	9.43	3.94	9.43
**OPL**	-1724.9731	-1724.3463	-393.2847	9.98	4.01	9.98
**B1LYP**	-1721.8422	-1721.2293	-384.5999	1.29	4.06	6.71
**MN12-L**	-1721.2561	-1720.6353	-389.5366	6.23	4.47	7.73
**M05-2X**	-1721.8997	-1721.2839	-386.4461	3.14	4.72	7.32
**B2GPPLYP**	-1720.5424	-1719.9409	-377.4683	-5.84	5.62	7.72
**wB97X-D**	-1721.8938	-1721.2770	-387.0849	3.78	6.26	18.95
**DSD-BLYP**	-1720.2703	-1719.6728	-374.9620	-8.34	7.67	10.01
**TPSSh**	-1721.9844	-1721.3805	-378.9655	-4.34	8.36	12.93
**SVWN**	-1717.9122	-1717.3157	-374.3151	-8.99	8.38	10.77
**BHandH**	-1717.4353	-1716.8450	-370.4113	-12.89	8.58	12.89
**M11**	-1721.7821	-1721.1542	-393.9787	10.67	8.86	10.67
**N12**	-1722.1646	-1721.5652	-376.0991	-7.21	9.11	15.03
**HCTH407**	-1722.4598	-1721.8293	-395.6316	12.33	9.37	12.38
**BP86**	-1722.1770	-1721.5768	-376.6155	-6.69	10.13	16.22
**M06**	-1721.7371	-1721.1360	-377.1909	-6.11	10.14	13.25
**B97-1**	-1721.7671	-1721.1563	-383.2627	-0.04	10.66	21.39
**B3P86**	-1723.6148	-1722.9816	-397.3283	14.02	10.73	14.02
**B97-2**	-1722.0826	-1721.4725	-382.8567	-0.45	11.43	22.56
**M05**	-1721.7947	-1721.1926	-377.8272	-5.48	11.56	15.80
**B97-D3**	-1722.2479	-1721.6532	-373.2069	-10.10	11.80	17.05
**BPW91**	-1722.0625	-1721.4645	-375.2607	-8.04	12.10	18.50
**mPWB95**	-1722.1449	-1721.5512	-372.5995	-10.71	12.45	17.30
**BPBE**	-1721.8184	-1721.2217	-374.4261	-8.88	12.88	19.24
**OLYP**	-1722.1170	-1721.5202	-374.5146	-8.79	13.72	18.46
**G96LYP**	-1722.0408	-1721.4453	-373.6938	-9.61	14.04	20.73
**VSXC**	-1722.6714	-1722.0514	-389.0502	5.74	14.89	21.92
**OTPSS**	-1722.0034	-1721.4064	-374.5987	-8.71	15.57	23.19
**M06-L**	-1721.8861	-1721.2996	-368.0312	-15.27	19.81	25.13
**SPW91**	-1713.4622	-1712.9122	-345.0951	-38.21	31.96	38.21

Group B

Table 4 - Benchmarking of the density functionals for the complexes of group B.

**DF**	**$Fe(H_2O)_3(CH_3O^-)$**			
	**$E_{elec}^{Fe^{2+}}$ (Hartree)**	**$E_{elec}^{Fe^{3+}}$ (Hartree)**	**$\Delta E_{elec}^{Fe^{3+}/Fe^{2+}}$ (kcal/mol)**	**CCSD(T)/CBS - DF**
**BMK**	-1607.3619	-1606.9276	-272.5155	3.71
**B3LYP**	-1608.1247	-1607.6963	-268.8307	0.02
**wB97X-D**	-1607.9892	-1607.5675	-264.5988	-4.21
**BB1K**	-1608.0668	-1607.6359	-270.3982	1.59
**mPWB1K**	-1608.0707	-1607.6382	-271.4173	2.61
**mPW1N**	-1608.0240	-1607.5898	-272.4916	3.68
**mPW1B95**	-1608.1182	-1607.6909	-268.1204	-0.69
**mPW1K**	-1608.0166	-1607.5815	-273.0306	4.22
**MN12-L**	-1607.4415	-1607.0067	-272.8449	4.03
**M06-2X**	-1607.9161	-1607.4767	-275.7440	6.93
**B3PW91**	-1607.9349	-1607.5089	-267.3090	-1.50

**MN12-SX**	-1607.6945	-1607.2550	-275.7722	6.96
**PBE1PBE**	-1607.4800	-1607.0546	-266.9200	-1.89
**OVWN5**	-1610.7510	-1610.3224	-268.9707	0.16
**OPL**	-1610.7126	-1610.2828	-269.6923	0.88
**B1LYP**	-1607.9582	-1607.5333	-266.6256	-2.18
**M05-2X**	-1607.9794	-1607.5378	-277.1352	8.32
**M11-L**	-1608.0814	-1607.6428	-275.2213	6.41
**mPW2PLYP**	-1607.1697	-1606.7411	-268.9048	0.09
**B97-2**	-1608.2028	-1607.7824	-263.7786	-5.03
**B2PLYP**	-1607.1118	-1606.6852	-267.7062	-1.10
**B97-1**	-1607.8741	-1607.4592	-260.3455	-8.46
**M06**	-1607.8473	-1607.4273	-263.5621	-5.25
**TPSSh**	-1608.0908	-1607.6716	-263.0639	-5.75
**M11**	-1607.8633	-1607.4162	-280.5626	11.75
**B2GPPLYP**	-1606.8277	-1606.4012	-267.6497	-1.16
**SVWN**	-1604.5110	-1604.0828	-268.7015	-0.11
**BHandH**	-1604.1182	-1603.6980	-263.7040	-5.11
**BP86**	-1608.2754	-1607.8561	-263.0821	-5.73
**DSD-BLYP**	-1606.5926	-1606.1680	-266.4261	-2.38
**HCTH407**	-1608.5756	-1608.1603	-260.6009	-8.21
**N12**	-1608.3482	-1607.9341	-259.8115	-9.00
**B3P86**	-1609.4873	-1609.0400	-280.6837	11.87
**M05**	-1607.9123	-1607.4994	-259.0579	-9.75
**BPW91**	-1608.1737	-1607.7585	-260.5212	-8.29
**VSXC**	-1608.6966	-1608.2782	-262.5600	-6.25
**BPBE**	-1607.9617	-1607.5477	-259.8052	-9.01
**mPWB95**	-1608.2586	-1607.8444	-259.8642	-8.95
**G96LYP**	-1608.1567	-1607.7457	-257.9478	-10.86
**B97-D3**	-1608.3867	-1607.9767	-257.2638	-11.55
**OTPSS**	-1608.1604	-1607.7536	-255.2439	-13.57
**M06-L**	-1607.9844	-1607.5780	-255.0117	-13.80
**OLYP**	-1608.2505	-1607.8437	-255.2433	-13.57
**SPW91**	-1600.6939	-1600.3047	-244.2380	-24.57

Table 4 (cont.)

DF	$Fe(H_2O)_3(CH_3S^-)$			
	$E_{elec}^{Fe^{2+}}$ (Hartree)	$E_{elec}^{Fe^{3+}}$ (Hartree)	$\Delta E_{elec}^{Fe^{3+}/Fe^{2+}}$ (kcal/mol)	CCSD(T)/CBS - DF
**BMK**	-1930.2562	-1929.8389	-261.8647	-1.24
**B3LYP**	-1931.1061	-1930.6879	-262.4728	-0.63
**wB97X-D**	-1930.9868	-1930.5688	-262.2858	-0.82
**BB1K**	-1931.1121	-1930.6997	-258.7768	-4.33
**mPWB1K**	-1931.1211	-1930.7082	-259.1326	-3.97
**mPW1N**	-1931.0397	-1930.6269	-259.0692	-4.04
**mPW1B95**	-1931.1623	-1930.7469	-260.6580	-2.45
**mPW1K**	-1931.0334	-1930.6210	-258.8037	-4.30
**MN12-L**	-1930.4387	-1930.0122	-267.6102	4.50
**M06-2X**	-1930.8917	-1930.4764	-260.5915	-2.52

**B3PW91**	-1930.8993	-1930.4814	-262.2337	-0.87
**MN12-SX**	-1930.6766	-1930.2544	-264.9590	1.85
**PBE1PBE**	-1930.3888	-1929.9736	-260.5376	-2.57
**OVWN5**	-1934.0743	-1933.6487	-267.1082	4.00
**OPL**	-1934.0334	-1933.6066	-267.7953	4.69
**B1LYP**	-1930.9427	-1930.5317	-257.8775	-5.23
**M05-2X**	-1930.9465	-1930.5316	-260.3537	-2.75
**M11-L**	-1931.0703	-1930.6372	-271.7562	8.65
**mPW2PLYP**	-1930.0834	-1929.6866	-249.0033	-14.10
**B97-2**	-1931.2257	-1930.8111	-260.1447	-2.96
**B2PLYP**	-1930.0139	-1929.6182	-248.2792	-14.83
**B97-1**	-1930.8346	-1930.4187	-260.9756	-2.13
**M06**	-1930.8320	-1930.4129	-262.9673	-0.14
**TPSSh**	-1931.0761	-1930.6600	-261.0847	-2.02
**M11**	-1930.8381	-1930.4225	-260.8111	-2.30
**B2GPPLYP**	-1929.7042	-1929.3136	-245.1284	-17.98
**SVWN**	-1926.8135	-1926.3751	-275.0657	11.96
**BHandH**	-1926.5390	-1926.1341	-254.0579	-9.05
**BP86**	-1931.2858	-1930.8657	-263.6080	0.50
**DSD-BLYP**	-1929.4430	-1929.0560	-242.8807	-20.23
**HCTH407**	-1931.6193	-1931.2029	-261.3376	-1.77
**N12**	-1931.3508	-1930.9338	-261.6407	-1.47
**B3P86**	-1932.6348	-1932.1953	-275.7842	12.68
**M05**	-1930.9235	-1930.5100	-259.4764	-3.63
**BPW91**	-1931.1647	-1930.7492	-260.7622	-2.35
**VSXC**	-1931.7951	-1931.3804	-260.2138	-2.89
**BPBE**	-1930.9200	-1930.5052	-260.2935	-2.81
**mPWB95**	-1931.2897	-1930.8747	-260.3869	-2.72
**G96LYP**	-1931.1514	-1930.7418	-257.0430	-6.06
**B97-D3**	-1931.4147	-1931.0039	-257.7614	-5.35
**OTPSS**	-1931.1527	-1930.7417	-257.9127	-5.19
**M06-L**	-1930.9587	-1930.5483	-257.5173	-5.59
**OLYP**	-1931.2545	-1930.8469	-255.8068	-7.30
**SPW91**	-1922.5305	-1922.1242	-254.9803	-8.13

Table 4 (cont.)

**DF**	**$Fe(H_2O)_3(NH_2CH_3)$**			
	**$E_{elec}^{Fe^{2+}}$ (Hartree)**	**$E_{elec}^{Fe^{3+}}$ (Hartree)**	**$\Delta E_{elec}^{Fe^{3+}/Fe^{2+}}$ (kcal/mol)**	**CCSD(T)/CBS - DF**
**BMK**	-1587.7429	-1587.0709	-421.6412	1.84
**B3LYP**	-1588.4984	-1587.8319	-418.2527	-1.55
**wB97X-D**	-1588.3854	-1587.7158	-420.1791	0.38
**BB1K**	-1588.4507	-1587.7802	-420.7326	0.93
**mPWB1K**	-1588.4544	-1587.7829	-421.4059	1.61
**mPW1N**	-1588.4206	-1587.7474	-422.3904	2.59
**mPW1B95**	-1588.4930	-1587.8267	-418.0857	-1.71
**mPW1K**	-1588.4145	-1587.7407	-422.8128	3.01
**MN12-L**	-1587.8121	-1587.1422	-420.3598	0.56

**M06-2X**	-1588.3001	-1587.6276	-422.0139	2.21
**B3PW91**	-1588.3214	-1587.6569	-416.9788	-2.82
**MN12-SX**	-1588.0696	-1587.3942	-423.8199	4.02
**PBE1PBE**	-1587.8734	-1587.2102	-416.1844	-3.62
**OVWN5**	-1591.1167	-1590.4503	-418.1604	-1.64
**OPL**	-1591.0788	-1590.4116	-418.6706	-1.13
**B1LYP**	-1588.3306	-1587.6669	-416.4850	-3.32
**M05-2X**	-1588.3602	-1587.6839	-424.4091	4.61
**M11-L**	-1588.4552	-1587.7830	-421.8150	2.02
**mPW2PLYP**	-1587.5664	-1586.9012	-417.4212	-2.38
**B97-2**	-1588.5889	-1587.9286	-414.2962	-5.50
**B2PLYP**	-1587.5090	-1586.8451	-416.6086	-3.19
**B97-1**	-1588.2646	-1587.6036	-414.8083	-4.99
**M06**	-1588.2173	-1587.5626	-410.8725	-8.93
**TPSSh**	-1588.4666	-1587.8106	-411.6713	-8.13
**M11**	-1588.2381	-1587.5560	-428.0443	8.24
**B2GPPLYP**	-1587.2335	-1586.5723	-414.9281	-4.87
**SVWN**	-1584.9390	-1584.2828	-411.8037	-8.00
**BHandH**	-1584.5503	-1583.8947	-411.3758	-8.42
**BP86**	-1588.6384	-1587.9878	-408.2432	-11.56
**DSD-BLYP**	-1587.0028	-1586.3448	-412.8680	-6.93
**HCTH407**	-1588.9594	-1588.3084	-408.4961	-11.30
**N12**	-1588.7187	-1588.0680	-408.2934	-11.51
**B3P86**	-1589.8676	-1589.1820	-430.1791	10.38
**M05**	-1588.2808	-1587.6298	-408.4679	-11.33
**BPW91**	-1588.5407	-1587.8930	-406.4003	-13.40
**VSXC**	-1589.0574	-1588.4119	-405.0806	-14.72
**BPBE**	-1588.3320	-1587.6856	-405.6353	-14.16
**mPWB95**	-1588.6071	-1587.9628	-404.2793	-15.52
**G96LYP**	-1588.5117	-1587.8674	-404.2937	-15.51
**B97-D3**	-1588.7680	-1588.1246	-403.7490	-16.05
**OTPSS**	-1588.5500	-1587.9073	-403.2809	-16.52
**M06-L**	-1588.3590	-1587.7186	-401.8665	-17.93
**OLYP**	-1588.6223	-1587.9804	-402.7695	-17.03
**SPW91**	-1581.1466	-1580.5315	-385.9698	-33.83

Table 4 (cont.)

**DF**	**$Fe(H_2O)_3(HCOO^-)$**				**Group B**	
	**$E_{elec}^{Fe^{2+}}$ (Hartree)**	**$E_{elec}^{Fe^{3+}}$ (Hartree)**	**$\Delta E_{elec}^{Fe^{3+}/Fe^{2+}}$ (kcal/mol)**	**CCSD(T)/CBS - DF**	**MUE**	**MaxE**
**BMK**	-1681.4250	-1680.9445	-301.5007	-1.30	2.02	3.71
**B3LYP**	-1682.2043	-1681.7317	-296.5817	-6.22	2.10	6.22
**wB97X-D**	-1682.0558	-1681.5795	-298.8985	-3.90	2.33	4.21
**BB1K**	-1682.1301	-1681.6522	-299.8749	-2.92	2.44	4.33
**mPWB1K**	-1682.1338	-1681.6544	-300.8720	-1.93	2.53	3.97
**mPW1N**	-1682.0775	-1681.5954	-302.5280	-0.27	2.65	4.04
**mPW1B95**	-1682.1894	-1681.7164	-296.7756	-6.02	2.72	6.02
**mPW1K**	-1682.0687	-1681.5857	-303.0978	0.30	2.96	4.30

**MN12-L**	-1681.4806	-1681.0026	-299.9596	-2.84	2.98	4.50
**M06-2X**	-1681.9856	-1681.5020	-303.4297	0.63	3.07	6.93
**B3PW91**	-1681.9845	-1681.5135	-295.5689	-7.23	3.11	7.23
**MN12-SX**	-1681.7368	-1681.2524	-303.9292	1.13	3.49	6.96
**PBE1PBE**	-1681.4928	-1681.0217	-295.5858	-7.21	3.82	7.21
**OVWN5**	-1685.0288	-1684.5615	-293.2484	-9.55	3.84	9.55
**OPL**	-1684.9857	-1684.5176	-293.7454	-9.05	3.94	9.05
**B1LYP**	-1682.0274	-1681.5572	-295.0845	-7.71	4.61	7.71
**M05-2X**	-1682.0630	-1681.5755	-305.8557	3.06	4.69	8.32
**M11-L**	-1682.1352	-1681.6571	-300.0129	-2.79	4.97	8.65
**mPW2PLYP**	-1681.1582	-1680.6829	-298.2641	-4.53	5.28	14.10
**B97-2**	-1682.2586	-1681.7932	-292.0599	-10.74	6.06	10.74
**B2PLYP**	-1681.0956	-1680.6221	-297.1559	-5.64	6.19	14.83
**B97-1**	-1681.9481	-1681.4805	-293.4203	-9.38	6.24	9.38
**M06**	-1681.9044	-1681.4429	-289.5825	-13.22	6.88	13.22
**TPSSh**	-1682.1689	-1681.7069	-289.8937	-12.90	7.20	12.90
**M11**	-1681.9347	-1681.4411	-309.7312	6.93	7.31	11.75
**B2GPPLYP**	-1680.7816	-1680.3092	-296.4725	-6.33	7.58	17.98
**SVWN**	-1678.2292	-1677.7655	-290.9485	-11.85	7.98	11.96
**BHandH**	-1677.8217	-1677.3543	-293.2728	-9.53	8.03	9.53
**BP86**	-1682.3584	-1681.9026	-286.0182	-16.78	8.64	16.78
**DSD-BLYP**	-1680.5233	-1680.0534	-294.8504	-7.95	9.37	20.23
**HCTH407**	-1682.6192	-1682.1654	-284.7695	-18.03	9.83	18.03
**N12**	-1682.4147	-1681.9625	-283.7592	-19.04	10.25	19.04
**B3P86**	-1683.6689	-1683.1766	-308.9072	6.11	10.26	12.68
**M05**	-1681.9748	-1681.5191	-285.9680	-16.83	10.39	16.83
**BPW91**	-1682.2506	-1681.7983	-283.8100	-18.99	10.76	18.99
**VSXC**	-1682.7917	-1682.3404	-283.1969	-19.60	10.87	19.60
**BPBE**	-1682.0187	-1681.5675	-283.1185	-19.68	11.42	19.68
**mPWB95**	-1682.3494	-1681.9009	-281.4487	-21.35	12.13	21.35
**G96LYP**	-1682.2370	-1681.7893	-280.9862	-21.81	13.56	21.81
**B97-D3**	-1682.4226	-1681.9748	-281.0151	-21.78	13.68	21.78
**OTPSS**	-1682.2020	-1681.7562	-279.7663	-23.03	14.58	23.03
**M06-L**	-1682.0594	-1681.6138	-279.6170	-23.18	15.13	23.18
**OLYP**	-1682.3170	-1681.8723	-279.0905	-23.71	15.40	23.71
**SPW91**	-1674.0698	-1673.6448	-266.7480	-36.05	25.64	36.05

Group C

Table 5 - Benchmarking of the density functionals for the complexes of group C.

**DF**	**$Fe(H_2O)_5(CH_3O^-)$**			
	**$E_{elec}^{Fe^{2+}}$ (Hartree)**	**$E_{elec}^{Fe^{3+}}$ (Hartree)**	**$\Delta E_{elec}^{Fe^{3+}/Fe^{2+}}$ (kcal/mol)**	**CCSD(T)/CBS - DF**
**mPW1B95**	-1761.0245	-1760.6414	-240.4045	-1.73
**PBE1PBE**	-1760.2827	-1759.9008	-239.6220	-2.51
**B3PW91**	-1760.8411	-1760.4585	-240.0694	-2.06
**wB97X-D**	-1760.9252	-1760.5388	-242.5098	0.38
**BB1K**	-1760.9647	-1760.5785	-242.3580	0.23

**mPWB1K**	-1760.9686	-1760.5810	-243.1806	1.05
**B3LYP**	-1761.0927	-1760.7075	-241.7217	-0.41
**B1LYP**	-1760.8641	-1760.4823	-239.6051	-2.53
**B97-1**	-1760.8014	-1760.4209	-238.7504	-3.38
**M06-2X**	-1760.8341	-1760.4406	-246.8999	4.77
**MN12-L**	-1760.2559	-1759.8672	-243.9192	1.79
**BMK**	-1760.2542	-1759.8646	-244.4513	2.32
**B97-2**	-1761.1119	-1760.7344	-236.8843	-5.25
**mPW1N**	-1760.9388	-1760.5489	-244.6264	2.49
**mPW1K**	-1760.9300	-1760.5395	-245.0450	2.91
**OVWN5**	-1764.2814	-1763.8944	-242.8179	0.69
**M05-2X**	-1760.9359	-1760.5405	-248.1380	6.01
**OPL**	-1764.2340	-1763.8460	-243.5214	1.39
**HCTH407**	-1761.5116	-1761.1383	-234.2744	-7.86
**mPW2PLYP**	-1759.8819	-1759.4982	-240.8287	-1.30
**BP86**	-1761.2378	-1760.8620	-235.7660	-6.37
**MN12-SX**	-1760.5504	-1760.1560	-247.4954	5.36
**B2PLYP**	-1759.8103	-1759.4280	-239.8824	-2.25
**M11-L**	-1761.0054	-1760.6118	-247.0122	4.88
**TPSSh**	-1761.0439	-1760.6365	-255.6424	13.51
**VSXC**	-1761.7461	-1761.3753	-232.6874	-9.44
**N12**	-1761.2180	-1760.8500	-230.9373	-11.19
**BPW91**	-1761.1135	-1760.7419	-233.1656	-8.97
**mPWB95**	-1761.1874	-1760.8174	-232.2068	-9.93
**BPBE**	-1760.8531	-1760.4826	-232.4490	-9.68
**M11**	-1760.7971	-1760.3957	-251.8647	9.73
**BHandH**	-1756.1632	-1755.7898	-234.3058	-7.83
**B97-D3**	-1761.2769	-1760.9096	-230.4755	-11.66
**G96LYP**	-1761.0727	-1760.7050	-230.7415	-11.39
**OTPSS**	-1761.0320	-1760.6679	-228.5107	-13.62
**M05**	-1760.7850	-1760.4355	-219.3152	-22.82
**DSD-BLYP**	-1759.1621	-1758.7839	-237.2902	-4.84
**M06**	-1760.7199	-1760.3783	-214.4049	-27.73
**OLYP**	-1761.1485	-1760.7844	-228.4825	-13.65
**B2GPPLYP**	-1759.4290	-1759.0754	-221.8761	-20.26
**B3P86**	-1762.8110	-1762.4073	-253.3124	11.18
**M06-L**	-1760.9315	-1760.5711	-226.1663	-15.97
**SVWN**	-1756.6993	-1756.3874	-195.7208	-46.41
**SPW91**	-1751.8890	-1751.6091	-175.5979	-66.53

Table 5 (cont.)

**DF**	**$Fe(H_2O)_5(CH_3S^-)$**			
	**$E_{elec}^{Fe^{2+}}$ (Hartree)**	**$E_{elec}^{Fe^{3+}}$ (Hartree)**	**$\Delta E_{elec}^{Fe^{3+}/Fe^{2+}}$ (kcal/mol)**	**CCSD(T)/CBS - DF**
**mPW1B95**	-2084.0622	-2083.6859	-236.1224	-3.26
**PBE1PBE**	-2083.1846	-2082.8087	-235.8965	-3.48
**B3PW91**	-2083.7980	-2083.4207	-236.7756	-2.60
**wB97X-D**	-2083.9125	-2083.5298	-240.1454	0.77

**BB1K**	-2084.0038	-2083.6295	-234.9088	-4.47
**mPWB1K**	-2084.0130	-2083.6379	-235.3939	-3.98
**B3LYP**	-2084.0662	-2083.6896	-236.3019	-3.08
**B1LYP**	-2083.8409	-2083.4709	-232.1490	-7.23
**B97-1**	-2083.7461	-2083.3712	-235.2790	-4.10
**M06-2X**	-2083.8038	-2083.4262	-236.9457	-2.43
**MN12-L**	-2083.2449	-2082.8649	-238.4411	-0.94
**BMK**	-2083.1406	-2082.7635	-236.5780	-2.80
**B97-2**	-2084.1276	-2083.7542	-234.3058	-5.07
**mPW1N**	-2083.9481	-2083.5730	-235.3575	-4.02
**mPW1K**	-2083.9404	-2083.5656	-235.1975	-4.18
**OVWN5**	-2087.5981	-2087.2152	-240.2659	0.89
**M05-2X**	-2083.8976	-2083.5205	-236.6294	-2.75
**OPL**	-2087.5482	-2087.1643	-240.9304	1.55
**HCTH407**	-2084.5510	-2084.1744	-236.3245	-3.05
**mPW2PLYP**	-2082.7892	-2082.4306	-225.0356	-14.34
**BP86**	-2084.2394	-2083.8623	-236.6727	-2.71
**MN12-SX**	-2083.5264	-2083.1455	-239.0177	-0.36
**B2PLYP**	-2082.7059	-2082.3485	-224.2524	-15.13
**M11-L**	-2083.9862	-2083.5947	-245.6637	6.28
**TPSSh**	-2084.0218	-2083.6474	-234.9188	-4.46
**VSXC**	-2084.8543	-2084.4719	-239.9803	0.60
**N12**	-2084.2167	-2083.8395	-236.7167	-2.66
**BPW91**	-2084.0962	-2083.7235	-233.8628	-5.52
**mPWB95**	-2084.2147	-2083.8381	-236.3213	-3.06
**BPBE**	-2083.8031	-2083.4311	-233.4643	-5.91
**M11**	-2083.7674	-2083.3901	-236.7625	-2.62
**BHandH**	-2078.5810	-2078.2096	-233.0495	-6.33
**B97-D3**	-2084.3025	-2083.9299	-233.8646	-5.51
**G96LYP**	-2084.0581	-2083.6925	-229.4294	-9.95
**OTPSS**	-2084.0178	-2083.6484	-231.8020	-7.58
**M05**	-2083.8099	-2083.4377	-233.5007	-5.88
**DSD-BLYP**	-2082.0063	-2081.6569	-219.2493	-20.13
**M06**	-2083.7309	-2083.3536	-236.7744	-2.60
**OLYP**	-2084.1455	-2083.7804	-229.1000	-10.28
**B2GPPLYP**	-2082.3263	-2081.9734	-221.3960	-17.98
**B3P86**	-2085.9505	-2085.5519	-250.1592	10.78
**M06-L**	-2083.8980	-2083.5308	-230.3688	-9.01
**SVWN**	-2079.0624	-2078.6669	-248.1624	8.78
**SPW91**	-2073.7805	-2073.4141	-229.8894	-9.49

Table 5 (cont.)

**DF**	**$Fe(H_2O)_5(NH_2CH_3)$**			
	**$E_{elec}^{Fe^{2+}}$ (Hartree)**	**$E_{elec}^{Fe^{3+}}$ (Hartree)**	**$\Delta E_{elec}^{Fe^{3+}/Fe^{2+}}$ (kcal/mol)**	**CCSD(T)/CBS - DF**
**mPW1B95**	-1741.4259	-1740.8305	-373.5821	0.86
**PBE1PBE**	-1740.7024	-1740.1094	-372.1169	-0.61
**B3PW91**	-1741.2545	-1740.6586	-373.9511	1.23

**wB97X-D**	-1741.3412	-1740.7418	-376.1562	3.43
**BB1K**	-1741.3745	-1740.7778	-374.4098	1.68
**mPWB1K**	-1741.3782	-1740.7811	-374.6401	1.92
**B3LYP**	-1741.4943	-1740.8952	-375.9491	3.22
**B1LYP**	-1741.2641	-1740.6687	-373.6204	0.90
**B97-1**	-1741.2110	-1740.6176	-372.3761	-0.35
**M06-2X**	-1741.2441	-1740.6479	-374.1155	1.39
**MN12-L**	-1740.6575	-1740.0550	-378.0638	5.34
**BMK**	-1740.6618	-1740.0629	-375.8493	3.12
**B97-2**	-1741.5258	-1740.9330	-372.0328	-0.69
**mPW1N**	-1741.3609	-1740.7620	-375.8393	3.11
**mPW1K**	-1741.3534	-1740.7544	-375.8951	3.17
**OVWN5**	-1744.6784	-1744.0693	-382.2047	9.48
**M05-2X**	-1741.3431	-1740.7439	-376.0457	3.32
**OPL**	-1744.6317	-1744.0217	-382.7896	10.06
**HCTH407**	-1741.9255	-1741.3351	-370.4954	-2.23
**mPW2PLYP**	-1740.3050	-1739.7148	-370.3869	-2.34
**BP86**	-1741.6282	-1741.0427	-367.3773	-5.35
**MN12-SX**	-1740.9556	-1740.3499	-380.1026	7.38
**B2PLYP**	-1740.2339	-1739.6442	-370.0285	-2.70
**M11-L**	-1741.4100	-1740.8026	-381.1411	8.42
**TPSSh**	-1741.4467	-1740.8592	-368.7058	-4.02
**VSXC**	-1742.1446	-1741.5626	-365.2105	-7.51
**N12**	-1741.6188	-1741.0344	-366.7153	-6.01
**BPW91**	-1741.5084	-1740.9253	-365.8606	-6.86
**mPWB95**	-1741.5641	-1740.9844	-363.7786	-8.95
**BPBE**	-1741.2511	-1740.6693	-365.0424	-7.68
**M11**	-1741.1998	-1740.5913	-381.8125	9.09
**BHandH**	-1736.6189	-1736.0446	-360.3749	-12.35
**B97-D3**	-1741.6881	-1741.1090	-363.3845	-9.34
**G96LYP**	-1741.4560	-1740.8755	-364.2724	-8.45
**OTPSS**	-1741.4503	-1740.8691	-364.6759	-8.05
**M05**	-1741.2039	-1740.6185	-367.3554	-5.37
**DSD-BLYP**	-1739.5984	-1739.0175	-364.5523	-8.17
**M06**	-1741.1531	-1740.5674	-367.5756	-5.15
**OLYP**	-1741.5498	-1740.9688	-364.5918	-8.13
**B2GPPLYP**	-1739.8880	-1739.3032	-366.9519	-5.77
**B3P86**	-1743.2179	-1742.6017	-386.6557	13.93
**M06-L**	-1741.3362	-1740.7636	-359.3251	-13.40
**SVWN**	-1737.2224	-1736.6385	-366.4417	-6.28
**SPW91**	-1732.4265	-1731.8881	-337.8216	-34.90

Table 5 (cont.)

**DF**	**$Fe(H_2O)_5(HCOO^-)$**				**Group C**	
	**$E_{elec}^{Fe^{2+}}$ (Hartree)**	**$E_{elec}^{Fe^{3+}}$ (Hartree)**	**$\Delta E_{elec}^{Fe^{3+}/Fe^{2+}}$ (kcal/mol)**	**CCSD(T)/CBS - DF**	**MUE**	**MaxE**
**mPW1B95**	-1835.1068	-1834.6785	-268.7874	0.74	1.65	3.26
**PBE1PBE**	-1834.3051	-1833.8783	-267.7972	-0.25	1.71	3.48

**B3PW91**	-1834.9005	-1834.4718	-269.0660	1.02	1.73	2.60
**wB97X-D**	-1834.9941	-1834.5615	-271.4349	3.39	1.99	3.43
**BB1K**	-1835.0391	-1834.6091	-269.8046	1.76	2.03	4.47
**mPWB1K**	-1835.0428	-1834.6121	-270.2458	2.20	2.29	3.98
**B3LYP**	-1835.1827	-1834.7510	-270.8651	2.82	2.38	3.22
**B1LYP**	-1834.9437	-1834.5157	-268.6174	0.57	2.81	7.23
**B97-1**	-1834.8726	-1834.4510	-264.5850	-3.46	2.82	4.10
**M06-2X**	-1834.9146	-1834.4825	-271.0973	3.05	2.91	4.77
**MN12-L**	-1834.3088	-1833.8751	-272.1540	4.11	3.04	5.34
**BMK**	-1834.3288	-1833.8954	-271.9745	3.93	3.04	3.93
**B97-2**	-1835.1778	-1834.7535	-266.2648	-1.78	3.20	5.25
**mPW1N**	-1835.0018	-1834.5688	-271.7399	3.69	3.33	4.02
**mPW1K**	-1834.9917	-1834.5585	-271.8616	3.82	3.52	4.18
**OVWN5**	-1838.5687	-1838.1347	-272.3379	4.29	3.84	9.48
**M05-2X**	-1835.0308	-1834.5952	-273.3243	5.28	4.34	6.01
**OPL**	-1838.5167	-1838.0817	-272.9309	4.89	4.47	10.06
**HCTH407**	-1835.5645	-1835.1459	-262.6642	-5.38	4.63	7.86
**mPW2PLYP**	-1833.8809	-1833.4566	-266.2234	-1.82	4.95	14.34
**BP86**	-1835.3300	-1834.9114	-262.6410	-5.40	4.96	6.37
**MN12-SX**	-1834.6055	-1834.1676	-274.7946	6.75	4.96	7.38
**B2PLYP**	-1833.8045	-1833.3811	-265.6725	-2.37	5.61	15.13
**M11-L**	-1835.0710	-1834.6345	-273.9129	5.87	6.36	8.42
**TPSSh**	-1835.1317	-1834.7113	-263.8313	-4.21	6.55	13.51
**VSXC**	-1835.8609	-1835.4497	-258.0576	-9.99	6.89	9.99
**N12**	-1835.2957	-1834.8814	-259.9559	-8.09	6.99	11.19
**BPW91**	-1835.1998	-1834.7846	-260.5037	-7.54	7.22	8.97
**mPWB95**	-1835.2888	-1834.8769	-258.4661	-9.58	7.88	9.93
**BPBE**	-1834.9193	-1834.5053	-259.7544	-8.29	7.89	9.68
**M11**	-1834.8814	-1834.4363	-279.2631	11.22	8.16	11.22
**BHandH**	-1829.8761	-1829.4633	-259.0365	-9.01	8.88	12.35
**B97-D3**	-1835.3230	-1834.9120	-257.8932	-10.15	9.17	11.66
**G96LYP**	-1835.1626	-1834.7507	-258.4850	-9.56	9.84	11.39
**OTPSS**	-1835.0822	-1834.6727	-256.9877	-11.06	10.08	13.62
**M05**	-1834.8805	-1834.4635	-261.6677	-6.38	10.11	22.82
**DSD-BLYP**	-1833.1033	-1832.6883	-260.4058	-7.64	10.20	20.13
**M06**	-1834.8226	-1834.4046	-262.2776	-5.77	10.31	27.73
**OLYP**	-1835.2241	-1834.8148	-256.8742	-11.17	10.81	13.65
**B2GPPLYP**	-1833.4205	-1833.0017	-262.7960	-5.25	12.32	20.26
**B3P86**	-1837.0024	-1836.5527	-282.1791	14.13	12.51	14.13
**M06-L**	-1835.0187	-1834.6159	-252.7771	-15.27	13.41	15.97
**SVWN**	-1830.4920	-1830.0743	-262.1295	-5.92	16.85	46.41
**SPW91**	-1825.3312	-1824.9551	-236.0182	-32.03	35.74	66.53
